# In Vitro Evidence of Selective Pro-Apoptotic Action of the Pure Cannabidiol and Cannabidiol-Rich Extract

**DOI:** 10.3390/molecules28237887

**Published:** 2023-12-01

**Authors:** Paweł Śledziński, Agnieszka Nowak-Terpiłowska, Piotr Rzymski, Ryszard Słomski, Joanna Zeyland

**Affiliations:** 1 Department of RNA Structure and Function, Institute of Bioorganic Chemistry, Polish Academy of Sciences, Noskowskiego 12/14, 61-704 Poznan, Poland; 2 Department of Biochemistry and Biotechnology, Poznań University of Life Sciences, Dojazd Street 11, 60-632 Poznan, Poland; joanna.zeyland@up.poznan.pl; 3 Department of Environmental Medicine, Poznań University of Medical Sciences, 60-806 Poznan, Poland; rzymskipiotr@ump.edu.pl; 4 Institute of Human Genetics, Polish Academy of Sciences, Strzeszyńska Street 32, 60-479 Poznan, Poland; slomski@up.poznan.pl

**Keywords:** cannabinoids, cannabidiol, CBD, *Cannabis*, cancer, apoptosis

## Abstract

Plant cannabinoids, secondary metabolites of species belonging to the *Cannabis* genus, can mimic the endocannabinoids’ action and exert biological effects. Considering the contribution of the endocannabinoid system in cell cycle and apoptotic regulation, there is an interest in exploring the potential anti-cancer activities of natural and synthetic cannabinoids. Cannabidiol (CBD), an abundant plant cannabinoid, reveals a low affinity to cannabinoid receptors and, contrary to various cannabinoids, lacks psychoactive action. Here, we present the in vitro assessment of the pro-apoptototic potential of CBD-rich extracts of *Cannabis sativa* L. (eCBD) compared to purified CBD (pCBD). As demonstrated, both eCBD and pCBD decreased the viability of breast cancer cell line MDA-MB-231 and human prostate cancer cell line PC-3 in a concentration-dependent fashion. Endoplasmic reticulum stress-related apoptosis and morphological changes were induced only in low-serum conditions. Moreover, the effects of eCDB and pCDB were also assessed in non-malignant cell lines (MCF-10A and PNT2) with no alterations of viability noted, ultimately suggesting a selective action of CBD in tumor cells. The results suggest the possible involvement of reactive oxygen species in the response mechanism to eCBD and pCBD, but no clear pattern was observed. We also demonstrated significant changes in gene expression involved in apoptosis and cell cycle control upon extract treatment. Altogether, our study shows the potential of eCBD and pCBD as novel pro-apoptototic agents that can be considered promising in future preclinical and clinical testing.

## 1. Introduction

An interest in the potential use of derivates of plant species belonging to the *Cannabis* genus has increased considerably in recent decades [1,2]. Various in vitro experiments, in vivo preclinical investigations, and clinical studies indicate that some secondary metabolites of these plants reveal promising activity for use not only in the palliative care of cancer patients as pain-relievers [3,4,5] but also in directly targeting the malignant cells and decreasing their viability [6,7,8,9]. Particular interest is paid to cannabinoids, lipophilic terpenophenolic molecules acting as ligands of a specific group of membrane G-protein-coupled receptors, known as cannabinoid receptors (CB receptors; CB1 and CB2), which, together with their endogenous ligands, constitute the endocannabinoid system (ES) [10]. Three main types of cannabinoids are recognized: (i) plant cannabinoids (phytocannabinoids), (ii) endocannabinoids, and (iii) synthetic cannabinoids. Plant cannabinoids are 21- or 22-carbon terpene phenolic secondary metabolites of plants belonging to the *Cannabis* genus, particularly *Cannabis sativa* L. [11].

More than 100 phytocannabinoids have been identified in *C*. *sativa* [12]. The most abundant include tetrahydrocannabinol (THC), which is responsible for the psychoactive effects of marijuana and hashish due to its high affinity for/to the CB1 receptor located in the central nervous system. However, it also displays immunomodulatory properties as it interacts with the CB2 receptor of the immune system cells [13]. The second well-recognized phytocannabinoid is cannabidiol (CBD). It does not display psychoactive properties since its affinity to CB1 receptors is low [13,14]. It also has little binding affinity to the CB2 receptor, although it can antagonize both in the presence of THC [15]. On the other hand, CBD interacts with other receptors such as transient receptor potential cation channels (TRPV1, TRPM8), orphan G protein-coupled receptors (GPR55, GPR119), and peroxisome proliferator-activated receptors (PPARs) [13]. The research conducted to date indicates that cannabinoids exhibit a range of promising anti-cancer properties, including antiproliferative, antiangiogenetic, and pro-apoptotic action [16,17,18,19,20]. In the case of THC, these effects were partially attributed to its stimulation of CB1 and CB2 receptors. The mechanisms of the anti-cancer action of CBD are less elucidated. One of the proposed models indicates the stimulation of reactive oxygen species (ROS) generation in a cell, which ultimately leads to autophagy and apoptosis [21,22,23,24]. Another model builds on the observations that CBD can block the degradation of N-arachidonoylethanolamine (AEA) by the inhibition of fatty acid amide hydrolase (FAAH) [25,26]. The observed effects may therefore be a result of increased AEA concentration. Other reports suggest the contribution of non-CB receptors, such as TRPM8, or indirect interaction with the CB2 receptor [13,27,28]. CBD has been demonstrated to decrease the viability of cancer cells originating from breast, lung, colorectal, or prostate cancer, neuroblastoma, glioblastoma, melanoma, and leukemia [17,23,24,27,29,30,31,32].

One should note that *Cannabis* also contains other molecules that may exert anti-tumorigenic actions. Therefore, the biomedical properties of *C*. *sativa* extracts may arise from interactions between particular constituents. These interactions may include mechanisms such as bioavailability or cellular transport regulation, metabolite activation or inactivation, and ligand–receptor interaction interference [12]. Moreover, compounds such as terpenes and flavonoids have been shown to act synergistically with cannabinoids. For example, terpenes can impact THC pharmacokinetics by increasing the blood–brain barrier’s permeability and the absorption of transdermal administration [12,33]. They can also impact the THC-CB1 affinity [34]. All in all, it would be of interest to compare the anti-cancer effects of cannabinoid-rich extracts with those of purified molecules.

To this end, the present study aimed to evaluate the effects of CBD-rich *C. sativa* extracts and purified CBD on the viability, morphology, gene expression, and apoptotic activity in two cancer cell lines and corresponding non-malignant cells. This allowed the identification of concentration ranges that induce apoptosis in the cancer cells but do not affect the normal cells. We conducted parallel experiments using pure cannabinoids and *C. sativa* botanical extracts in our study to better understand the effects of individual cannabinoids versus complex mixtures present in extracts. The study also aimed to assess the contribution of ROS in the pro-apoptototic effects of CBD and CBD-rich extracts.

## 2. Results

### 2.1. Cell Viability

The study was initiated with a panel of cell lines of various origins and proliferative potentials. Two cancer cell lines, MDA-MB-231 (breast cancer) and PC-3 (prostate cancer), and their two normal counterparts, MCF-10A (mammary gland epithelium) and PNT2 (prostate epithelium), were employed. Additionally, two widely studied cell lines of distinct origin were used: MSU-1.1 (v-myc-transformed human fibroblasts) and HEK-293 (human embryonic kidney cells). Cells were treated with pure CBD at a concentration ranging from 0 to 15 µM, or with two *C. sativa* extract solutions (extract B and extract D, see Table 1 for detailed characteristics) containing equimolar CBD concentrations. Two experimental settings were applied: full-fetal bovine serum (FBS) medium (10% FBS in the medium) and low-FBS medium (0.5% FBS in the medium). The presence of serum in cell culture media can significantly influence cell behavior. Serum contains various growth factors, hormones, and proteins that may interact with cannabinoids, potentially affecting their bioavailability and cellular responses.

**Figure 1:**
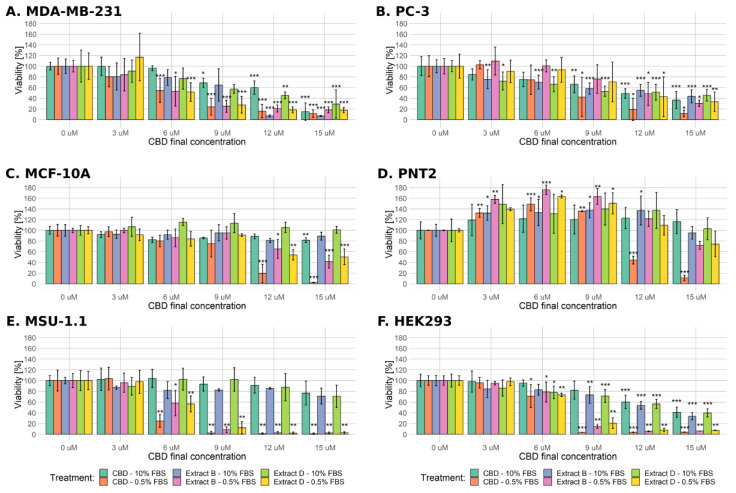
Changes in the viability of the studied cell lines upon treatment with pure CBD or CBD-rich *C*. *sativa* extracts. Results obtained for every studied solution in both serum conditions are combined into separate plots for every cell line used. The numbers represent a percentage (mean ± SD) of the untreated sample viability. Treatment in low-FBS conditions elicited a more robust response, especially in the case of pure CBD solution. (**A**) MDA-MB-231 cells, (**B**) PC-3 cells, (**C**) MCF-10A cells, (**D**) PNT2 cells, (**E**) MSU-1.1 cells, (**F**) HEK293 cells. ***—*p* < 0.001; **—*p* < 0.01; and *—*p* < 0.05.

The overall cell response was less profound in the standard medium conditions than in the low-FBS conditions (Figure 1). In the case of the MDA-MB-231 cell line cultivated in standard medium conditions, a significant decrease in viability was observed at the CBD concentration ≥ 9 µM for the pure CBD solution and 12 µM for extracts B and D (Figure 1, left upper panel). At the highest concentration (15 µM CBD), MDA-MB-231 cells exhibited the most robust response among all cell lines used. All of the studied mixtures in the low-FBS medium conditions significantly decreased MDA-MB-231 cell viability at the CBD concentration of 6 µM or higher.

Similarly to MDA-MB-231 cells, the PC-3 cell line exhibited a significant decrease in viability at the CBD concentration ≥ 9 µM for the pure cannabidiol solution in standard medium conditions (Figure 1 right upper panel). Concentration 3 µM CBD in both extracts was high enough to induce a significant decrease in the viability of PC-3 cells, but the response to the highest CBD concentration in all of the studied mixtures was less profound than in MDA-MB-231 cells (a decrease to approximately 40%). Surprisingly, PC-3 cells turned out to be much more resistant in low-FBS conditions as the significant decrease in viability was only observed at ≥9 µM CBD concentration in the case of pure CBD solution and at the two highest concentrations of CBD in both of the extract solutions used (12 and 15 µM CBD).

The viability of non-cancerous MCF-10A cells in the standard medium was unchanged or slightly decreased (significant only after treatment with 15 µM pure CBD solution, Figure 1). Significantly decreased viability was observed only at the two highest concentrations of CBD in all of the studied mixtures in low-FBS conditions. The most profound reaction was observed for the highest concentration of pure CBD solution (a decrease of viability to 2.4%).

The PNT2 cells mainly responded oppositely as the observed viability decrease was significant only for the two highest concentrations of pure CBD in low-FBS conditions. In contrast, both extract studies elicited only slight viability reduction in the highest concentration used (Figure 1). Interestingly, this cell line reacted with a considerable increase in viability in low and intermediate CBD concentrations in all experimental settings (3–9 µM CBD).

MSU-1.1 and HEK-293 turned out to be the most sensitive cells in the low-FBS conditions as 6 µM CBD concentration in all mixtures was enough to decrease the viability in a significant way. For the concentration of 9 µM CBD or higher, the observed viability did not exceed 20%. Similarly to MCF-10A, MSU-1.1 cells were relatively resistant in standard medium conditions as no significant decrease in viability was observed (Figure 1). The decrease in the viability of HEK-293 cells in standard medium conditions was comparable to the PC-3 cell line (Figure 1).

Based on obtained data, IC50 concentrations were calculated for the studied solutions and are summarized in Table 2.

Since the MSU-1.1 and HEK-293 cell lines are characterized by different origins and proliferative potential than the rest of the cells used, and since they are not a relevant model in cancer studies, we excluded them from further experiments.

### 2.2. Cell Morphology

**Figure 2:**
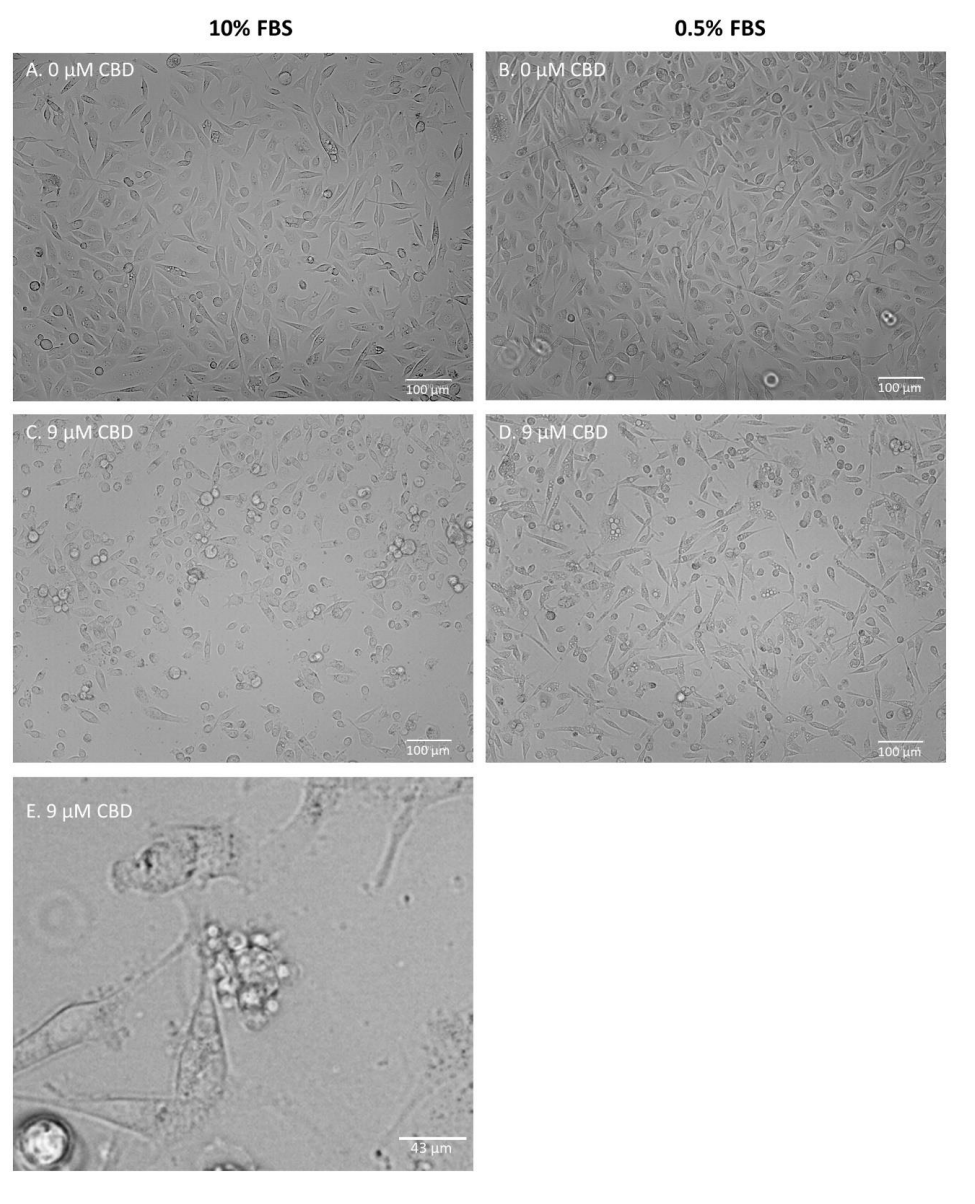
Representative images of PC-3 cells after 24 h of incubation with extract B. (**A**,**B**)—control samples; (**C**,**D**)—samples treated with extract B containing 9 µM CBD. Cells were incubated in either standard (**A**,**C**) or low-FBS (**B**,**D**) medium conditions. (**E**)—zoomed photo depicting the formation of apoptotic bodies in MDA-MB-231 cells after 24 h of incubation with extract D (9 µM CBD) in low-FBS medium conditions (see: Supplementary Figure S3).

Figure 2 and Figures S1–S12 present representative images of the cells after 24 h of incubation, with the studied mixtures in either standard or low-FBS medium conditions. MDA-MB-231 cells did not exhibit any significant changes in morphology after incubation, with all of the studied mixtures containing 9 µM CBD or 9 µM CBD extracts in a standard medium. Moreover, 15 µM pure CBD solution and 15 µM CBD extracts induced mostly cell detachment and decreased the number of visible cells, suggesting proliferation inhibition (Supplementary Figures S1–S3). Cell shrinking and rounding were also observed. In the low-serum medium, morphological changes such as shrinking, the formation of apoptotic bodies, and cell detachment were observed. The described changes were also observed in cells incubated with 9 µM CBD extracts and in pure 9 µM CBD (Supplementary Figures S1–S3). PC-3 cells responded similarly (Figure 2 and Figures S4–S6). In standard medium conditions, morphological changes were limited. The main response included partial cell detachment and a decrease in cell number. Again, the low-FBS serum was required to induce substantial morphological changes, suggesting apoptotic cell death. The formation of apoptotic bodies was even more visible than in MDA-MB-231 cells (Figure 2E). We did not observe any morphological changes in PNT2 cells regardless of the solution analyzed (Supplementary Figures S7–S9). The only response was a decrease in cell number in the case of 15 µM CBD solution. MCF-10A cells did not exhibit any response in the standard medium condition. In the case of a low-FBS medium, a profound cell reaction to all of the studied mixtures was observed at the highest concentration used (Supplementary Figures S10–S12).

These results suggest that a low-FBS medium is required to induce clear changes in cell morphology that can be ascribed to the process of apoptosis. Therefore, in the next steps we verified the hypothesis that low-FBS medium conditions are necessary to induce apoptosis in cancer cells. 

### 2.3. Apoptotic Activity

To analyze in detail the apoptotic activity of cells exposed to CBD and CBD-rich C.sativa extracts, three complementing methods were applied, namely, the luminometric assay, the Muse Caspase 3/7 kit, and flow cytometry. We started with a ‘bulk’ method based on examining the entire population of cells and then moved to ‘single-cell’ methods, which allowed for more precise measurements. Each apoptosis assay provides unique insights into different aspects of the apoptotic pathway, allowing for a more thorough assessment of the phenomenon.

#### 2.3.1. Luminometric Caspase 3/7 Assay

At this stage, we decided to narrow the range of concentrations used. The earlier experiments demonstrated that the lowest CBD concentration (3 µM CBD) does not lead to any significant decrease in the viability of cancer cells, whereas the highest (15 µM CBD) elicits a strong response in cancerous or non-malignant cells. Thus, solutions containing 0, 6, 9, and 12 µM CBD were employed in the next experiment. A luminometric assay assessed the activity level of executive caspases 3/7.

**Figure 3:**
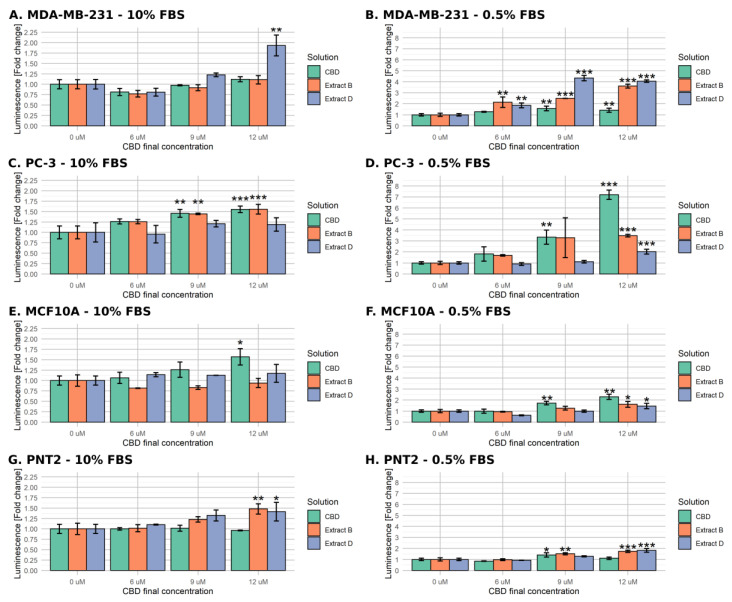
Changes in caspase 3/7 activity in cells upon treatment with pure CBD or *C. sativa* extracts measured by luminometric assay. The numbers represent a proportion (mean ± SD) of the untreated sample luminescence. Left panel (**A**,**C**,**E**,**G**)—treatment in standard medium conditions. Right panel (**B**,**D**,**F**,**H**)—treatment in low-FBS medium conditions. (**A**,**B**)—MDA-MB-231 cells. (**C**,**D**)—PC-3 cells. (**E**,**F**)—MCF10A cells. (**G**,**H**)—PNT2 cells. ***—*p* < 0.001; **—*p* < 0.01; and *—*p* < 0.05.

In standard medium conditions, the MDA-MB-231 cells exhibited a significant, almost two-fold increase in caspase activity only when incubated with 12 µM CBD extract D (Figure 3A). Conversely, in the case of PC-3 cells, CBD and extract B stimulated a significant increase (approximately 0.5-fold) in caspases activity at 9 µM and 12 µM CBD (Figure 3C). MCF-10A normal cells responded only for 12 µM CBD (Figure 3E). The treatment of PNT2 resulted in a significant response in the cases of extract B and extract D, at the highest concentration used (Figure 3G).

The low-FBS medium conditions enabled a much stronger response among all of the studied cell lines. As depicted in Figure 3B, the MDA-MB-231 cells’ response for CBD was significant only for 9 µM and 12 µM (an approximate 0.5-fold increase in caspases activity). However, it was remarkably more robust for both extracts in all of the concentrations used, reaching a 4-fold increase in luminescence for extract D. Conversely, in the case of PC-3 cells, Extract D turned out to be the least active, inducing a significant change only in the highest concentration used (2-fold increase, Figure 3D). The CBD treatment led to a notable increase in luminescence for either CBD and extract B at the concentration of 9 µM and to a very strong response for CBD at 12 µM.

The non-malignant cells responded in a less remarkable way. MCF-10A cells exhibited a significant increase of caspase activity for all mixtures at the highest CBD concentration used (up to 2.25-fold increase for CBD) and only for CBD at 9 µM concentration (1.75-fold increase, Figure 3F). The response of PNT2 cells was even weaker as it reached a 1.5-fold increase for 9 µM extract B and a 1.75-fold increase in the case of 12 µM extract B and D (Figure 3H). The luminometric assay results support the hypothesis that the low-FBS serum is necessary to induce the apoptosis of MDA-MB-231 and PC-3 cells by CBD and CBD-rich *C. sativa* extracts.

#### 2.3.2. Cell Analyzer Caspase-3/7 Assay

**Figure 4:**
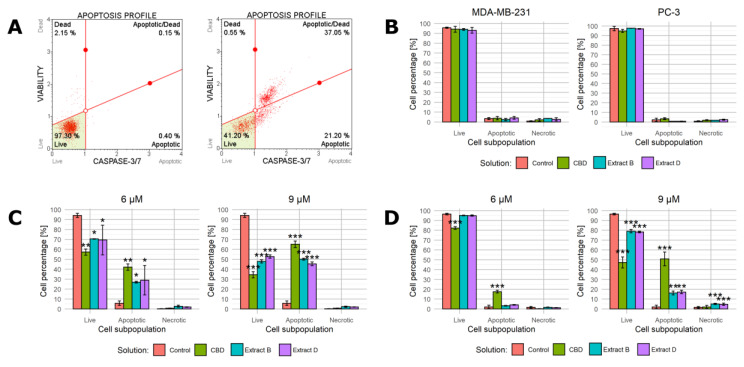
Changes in caspase 3/7 activity in cells upon treatment with pure CBD or *C. sativa* extracts measured by Muse™ Caspase-3/7 Kit. (**A**)—a representative example of results obtained for PC-3 cells incubated with studied solutions at 9 μM CBD concentration in the low-FBS medium for 24 h (right panel) and a control sample (left panel). (**B**)—results for MDA-MB-231 and PC-3 cells treated with studied solutions at concentrations of 9 µM CBD/CBD in extract in the complete medium. (**C**)—results for MDA-MB-231 cells treated with studied solutions at 6 µM and 9 µM CBD concentrations in the low-FBS medium. (**D**)—results for PC-3 cells treated with studied solutions at 6 µM and 9 µM CBD concentrations in the low-FBS medium. Data are presented as a percentage distribution of cells among subpopulations. Bars represent mean, and whiskers represent SD. CBD—cannabidiol, EB—extract B, and ED—extract D. ***—*p* < 0.001; **—*p* < 0.01; and *—*p* < 0.05.

At this stage, the range of concentrations was further narrowed. The luminometric assay demonstrated that the highest concentration (12 µM CBD) led to apoptotic activity in cancerous and non-malignant cells. In the concentration range of 6 and 9 μM, the average increase in the caspase 3/7 activity did not exceed 1.75 of the base value for the normal cells. Consistent with the previous observations (a lack of significant viability decrease or morphological changes), these results suggest that this concentration range is optimal to induce a significant response of cancer cells and a limited response of normal cells. Therefore, in the next experiment, solutions containing 0, 6, and 9 µM CBD were employed. The apoptotic activity was further analyzed based on caspase 3/7 activity detected by the Muse cell analyzer. Both luminometric and cell analyzer assays utilize the activity of caspases 3 and 7 as apoptosis markers, but the detection methods are different (bulk luminometric detection vs. single-cell fluorescence detection). The Muse Caspase-3/7 kit allows for a more detailed measurement of live, early, late apoptotic, total apoptotic, and dead cell percentages in the population. Figure 4A presents a representative example of the results obtained for PC-3 cells incubated with 9 μM CBD in the low-FBS medium for 24 h (right panel) and a control sample (left panel). The complete set of data is presented in Figure 4B–D.

In the first step, both cancer cell lines were treated with studied solutions containing 9 µM CBD/CBD in extract in the complete medium (Figure 4B). As expected, the complete medium conditions prevented apoptosis initiation. Regardless of the cell line and solution administrated, the percentage of apoptotic cells did not exceed 5%. The fraction of live cells was approximately 95% and 97% for MDA-MB-231 and PC-3 cells, respectively.

When the low-FBS medium conditions were applied, the outcomes were radically different. The MDA-MB-231 cells responded with a distinct shift in the cell distribution between subpopulations. Moreover, 6 µM CBD and extracts containing 6 µM CBD led to a statistically significant increase in the percentage of cells belonging to the apoptotic subpopulation (an increase to 42%, 26.9%, and 28.8%, respectively, Figure 4C, left panel). A proportional decrease in the percentage of living cells accompanied this change. The 9 µM concentration led to a similar but stronger response (Figure 4C, right panel). Interestingly, regardless of the concentration, the necrotic cell fraction percentage was unchanged. 

The response of PC-3 cells was different. When the cells were incubated with solutions containing 6 µM CBD, only CBD and not the extracts led to a statistically significant increase in the apoptotic cells’ percentage (Figure 4D, left panel). All of the solutions containing 9 µM CBD elicited a significant response, but extracts B and D turned out to be less potent (constituting an increase in the percentage of the apoptotic cells to 50.8%, 16.2%, and 17.1%, Figure 4D, right panel). Similarly to MDA-MB-231 cells, the increase in the necrotic fraction was limited. 

#### 2.3.3. Annexin V Assay

**Figure 5:**
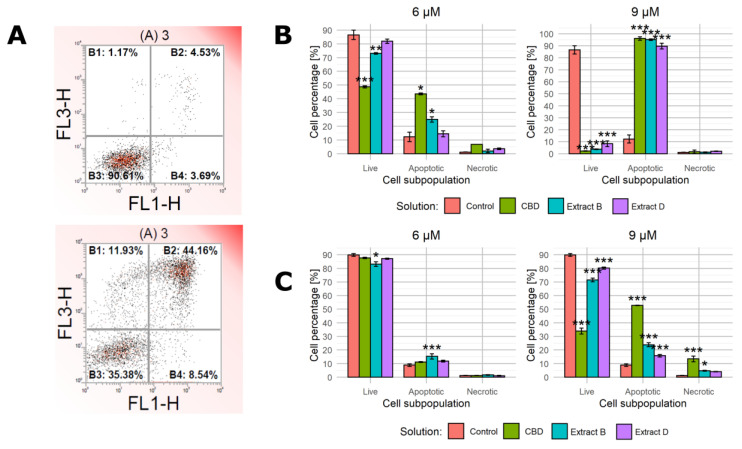
Apoptotic activity of cells upon treatment with pure CBD or *C. sativa* extracts measured by flow cytometry. (**A**)—a representative example of results obtained for PC-3 cells incubated with studied solutions at 9 μM CBD concentration in the low-FBS medium for 24 h (bottom panel) and a control sample (top panel). X-axis—fluorescence intensity of FIT-labelled annexin V. Y-axis—fluorescence intensity of propidium iodide. (**B**)—results for MDA-MB-231 cells treated with studied solutions at 6 µM and 9 µM CBD concentrations in the low-FBS medium. (**C**)—results for PC-3 cells treated with studied solutions at 6 µM and 9 µM CBD concentrations in the low-FBS medium. Data are presented as a percentage distribution of cells among subpopulations. Bars represent mean, and whiskers represent SD. CBD—cannabidiol, EB—extract B, and ED—extract D. ***—*p* < 0.001; **—*p* < 0.01; and *—*p* < 0.05.

Because the full-medium conditions led to no significant changes in the previous step, we decided to characterize the cell response only in the low-FBS conditions during this step. We used annexin V and propidium iodide staining followed by flow cytometry to characterize cell apoptotic activity in detail. Figure 5A presents a representative example of the results obtained for PC-3 cells incubated with 9 μM CBD in the low-FBS medium for 24 h (bottom panel) and a control sample (top panel). The complete set of data is presented in Figure 5B,C.

In the case of MDA-MB-231 cells incubated with solutions containing 6 µM CBD, only the pure CBD solution and extract B led to a significant shift in cell distribution among subpopulations (Figure 5B, left panel). The higher CBD concentration elicited a much stronger response to all of the solutions used (the apoptotic cells’ percentage increased to 96.1%, 95.1%, and 89.7% for CBD, extract B, and extract D, respectively, Figure 5B).

The response of PC-3 cells followed a different pattern. The treatment with 6 µM CBD solution and 6 µM CBD extracts led only to minor changes, with extract B being the only one that induced a statistically significant change (Figure 5C). The higher CBD concentration in solutions (9 µM CBD) led to a stronger response, especially in the case of a pure CBD solution (the apoptotic cells’ percentage was 52.8, 23.8%, and 75% for CBD, extract B, and extract D, respectively, Figure 5C, right panel). The response was, however, much less evident than in MDA-MB-231 cells in the corresponding conditions. Notably, the administration of 9 µM CBD solution was the setting that led to an apparent increase in the percentage of necrotic cells (an increase to 13.4%). 

### 2.4. ROS Involvement

**Figure 6:**
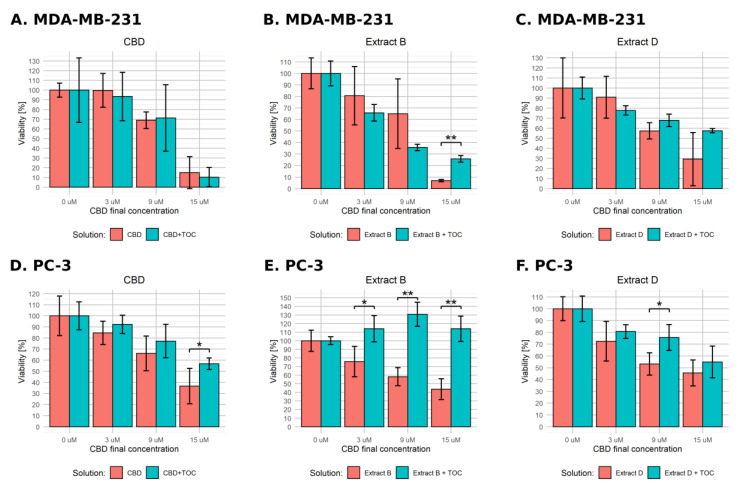
Changes in the viability of the studied cell lines upon treatment with pure CBD or *C. sativa* extract combined with α-tocopherol in standard medium conditions. The numbers represent a percentage (mean ± SD) of the untreated sample viability. (**A**–**C**)—MDA-MB-231 cells. (**D**–**F**)—PC-3 cells. TOC—α-tocopherol. **—*p* < 0.01; and *—*p* < 0.05.

Assuming the reactive oxygen species play an important role in regulating the apoptotic pathways, administering antioxidant substances such as α-tocopherol (α-TOC) should at least partially hamper their action. To verify the hypothesis of the ROS involvement in CBD pro-apoptototic action, we conducted an experiment corresponding to the earlier described viability assay, but we additionally administered α-TOC to the final concentration of 10 µM. We only used cancer cell lines and applied the normal- and low-FBS medium conditions and final CBD concentrations in solutions of 0, 3, 9, and 15 µM. The results generated with full medium conditions are presented in Figure 6. MDA-MB-231 cells exhibited a statistically significant difference between α-TOC-treated and untreated variants only in the case of extract B containing 15 µM CBD (viability level of 25.8% vs. 6.9%, Figure 6B). The PC-3 cells responded more distinctly. In every experimental setting, α-TOC administration decreased cell response (Figure 6D–F). In cases of pure CBD solution and extract D, the effect reached significance only for 15 and 9 µM, respectively (Figure 6D,F). The response was exceptionally high for the combination of α-TOC and extract B (Figure 6E). α-TOC administration caused not only a lack of cell viability decrease but even an increase of this parameter in every experimental setting (75.7% vs. 113.9%, 58.2% vs. 130.7%, and 43.6% vs. 113.9% for 3, 9, and 15 µM, respectively).

**Figure 7:**
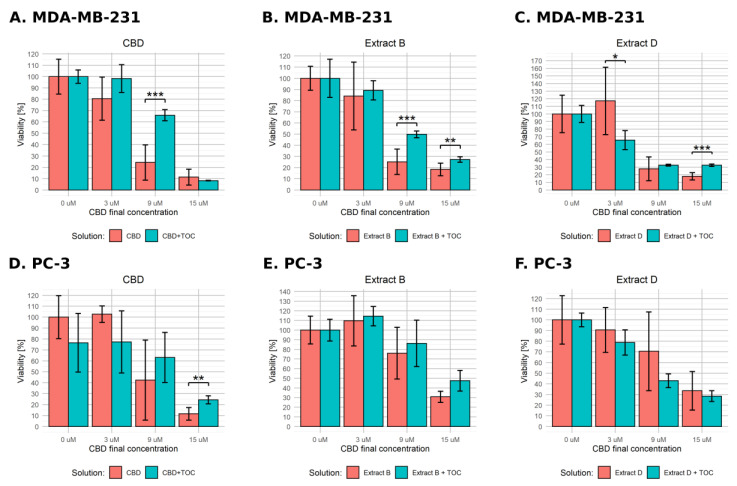
Changes in the viability of the studied cell lines upon treatment with pure CBD or *C. sativa* extract combined with α-tocopherol in low-FBS medium conditions. The numbers represent a percentage (mean ± SD) of the untreated sample viability. (**A**–**C**)—MDA-MB-231 cells. (**D**–**F**)—PC-3 cells. TOC—α-tocopherol. ***—*p* < 0.001; **—*p* < 0.01; and *—*p* < 0.05.

The results obtained in low-FBS medium conditions are presented in Figure 7. In the case of MDA-MB-231 cells incubated with pure CBD solution in combination with α-TOC, we observed a significant effect for 9 µM CBD (Figure 7A). For Cannabis extracts, α-TOC led to fewer distinct changes, but the observed effect reached significance in cases of extract B containing 9 µM CBD and 15 µM CBD (Figure 7B) and extract D containing 3 µM CBD and 15 µM CBD (Figure 7C). Contrary to expectations, the effect observed for 3 µM CBD was reversed—α-TOC led to a significant decrease in cell viability. PC-3 cells responded in a significant way only for the combination of α-TOC with the pure 15 µM CBD solution (11.6% vs. 24.3%, Figure 7D). We did not observe any significant effect in the other experimental settings. 

### 2.5. Changes in Gene Expression

Given the results of the previous experiments, in the further step, the gene expression profile in cancer cells and their non-malignant counterparts treated with CBD and *Cannabis* extracts was analyzed. Cancer cells were incubated with solutions at concentrations equal to IC50 calculated earlier (Table 2). In the case of MDA-MB-231 cells, the concentrations used were 6.04 µM, 6.01 µM, and 6.09 µM for CBD, extract B, and extract D, respectively. In the case of PC-3 cells, the concentrations used were 8.14 µM, 11.58 µM, and 10.98 µM for CBD, extract B, and extract D, respectively. Normal cells were treated with solutions at concentrations of IC50 calculated for their cancer counterparts. Genes of interest were selected based on a literature review concerning the impact of cannabinoids on cancer cells. *BBC3*, *DDIT3,* and *PTGS* gene products (PUMA, CHOP, and COX2, respectively) are involved in the regulation of the process of apoptosis. *CDKN1A* and *CDKN1B* genes encode cyclin-dependent kinase inhibitors, which regulate the cell cycle in response to various stress stimuli. To conduct the analysis, we used a quantitative PCR reaction.

**Figure 8:**
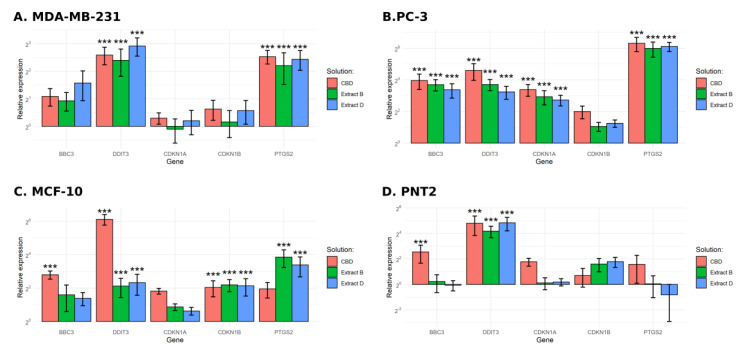
Results of gene expression analysis. (**A**)—MDA-MB-231 cells. (**B**)—PC-3 cells. (**C**)—MCF-10 cells. (**D**)—PNT2 cells. ***—*p* < 0.001.

In the case of MDA-MB-231 cells, a significant increase in the expression of *DDIT3* was observed for all of the solutions studied. The change observed was approximately 6-, 5-, and 7-fold increase for CBD, extract B, and extract D, respectively (Figure 8A). A similar pattern of expression was obtained for *PTGS2*. The expression of *BBC3* was slightly increased but did not reach statistical significance.

PC-3 cells exhibited a more substantial response (Figure 8B). We observed at least a 10-fold increase in the expression of *BBC3* and *DDIT3*, regardless of the solution used, including a 25-fold increase in the case of *DDIT3* after incubation with pure CBD. An exceptionally high increase in expression was observed for the *PTGS2* (more than 60-fold for all of the solutions studied). An increase in expression was also observed for the *CDKN1A*, ranging from a 5- to 10-fold increase. 

MCF-10 cells responded differently (Figure 8C). In the case of *BBC3*, only pure CBD elicited a significant response (more than a 6-fold increase). The *DDIT3* expression level increased more than 4-fold for extracts B and D and approximately 70-fold for pure CBD. Incubation with all of the studied solutions led to an approximate 4-fold increase in *CDKN1B* expression. The increase of *PTGS2* expression reached significance only for extracts B and D.

In the case of the PNT2 cell line, we observed a statistically significant increase in the expression of *DDIT3* (18-fold for extract B and 20-fold for pure CBD and extract D, Figure 8D). The increase in the expression level of *BBC3* reached significance only in the case of pure CBD treatment. 

## 3. Discussion

Breast cancer and prostate cancer are among the most prevalent types of cancer worldwide and have limited treatment options, especially for advanced or treatment-resistant cases. Exploring the potential of CBD as a novel or adjuvant therapy provides hope for patients who may have exhausted conventional treatment options. The present study demonstrated that CBD and CBD-rich *C. sativa* extracts decreased the viability of MDA-MB-231 and PC-3 cancer cells in a concentration-dependent fashion. In low-FBS medium conditions, all of the solutions studied led to a more profound effect than in full-FBS medium, but the pure CBD solution was the most potent agent. Low and intermediate concentrations (3–9 µM CBD/CBD in extract) were stimulatory in the case of the PNT2 normal cell line in low-FBS conditions. The comparison of the results from the viability assay and a luminometric assay of caspase activity allowed for the estimation of the concentration range of 6–9 µM pure CBD/CBD in the extract as optimal, leading to a significant response of cancer cells and no accompanying response of normal cells. The response consists of a decrease in cell viability and the initiation of apoptosis. The observations presented here support the view that cannabinoids act as promising pro-apoptototic agents exhibiting selectiveness toward cancer cells. They show, however, differential effects based on cancer type, as demonstrated by distinct apoptotic and gene expression profiles after treatment. Nevertheless, the results presented here provide a ground for further preclinical studies to determine the route of administration, safety dosing, and in vivo biological efficacy.

All of the studied solutions led to a substantial increase in the caspase activity; phosphatidylserine translocation; and evident changes in cancer cell morphology only in the low-FBS conditions. Again, the pure CBD solution had the highest potency, except for the luminometric assay of MDA-MB-231 cells, which demonstrated that extracts were more potent in that case. These results suggest that apoptosis is the key mechanism responsible for the decreased cancer cell viability in low-FBS conditions. The viability decrease observed in full medium conditions seems to rely on another mechanism. The proliferation inhibition observed upon morphology assessment suggests that the process responsible for the changes described above may be cell cycle arrest. CBD exposure may activate mechanisms leading to cell cycle arrest in the G0/G1 phase and eventually to proliferation inhibition. The verification of this hypothesis requires additional analyses. 

The role of FBS in the observed phenomena may result from the binding of cannabinoids to high-mass serum proteins, such as bovine serum albumin. By binding to cannabinoids, it may interfere with their interaction with membrane receptors or unknown intercellular targets. This hypothesis is supported by studies demonstrating the high affinity of cannabinoids to serum albumin [35,36]. On the other hand, the absence of serum growth and proliferation factors may lead to the higher vulnerability of cells to apoptosis-inducing stimuli. 

**Figure 9:**
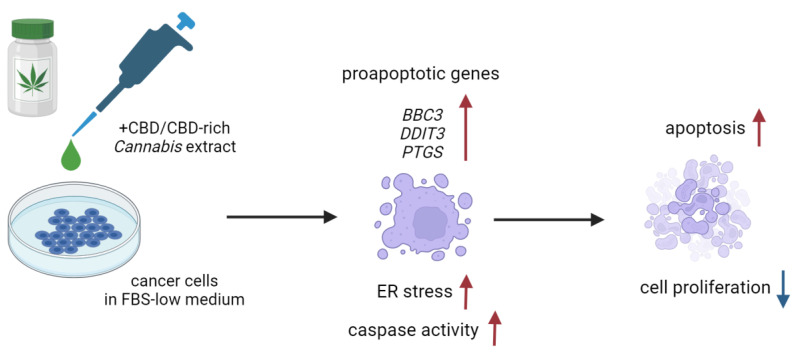
Possible mechanism of pro-apoptotic action of the CBD-rich *C. sativa* extracts. Created with BioRender.com.

The induction of apoptosis in studied cell lines was probably mediated by endoplasmic reticulum stress (ER stress), which is suggested by the increase in expression of its stress, *DDIT3* (Figure 9). In turn, its elevated expression leads to an increased *BBC3* mRNA level, observed in PC-3 cells and not in MDA-MB-231 cells. The results presented also suggest that the *PTGS2* gene plays a role in response to cannabinoid exposure. Indeed, the literature data point to its possible function in apoptosis induction by anandamide and its analogue R(+)-metananandamide [37,38,39]. Available data suggest that *PTGS2* expression may be stimulated by ceramide accumulation, in agreement with reports of its crucial role in the response of cancer cells to cannabinoids. In turn, PTGS2 synthase activity leads to the synthesis of prostaglandin activating PPAR receptors, which may stimulate apoptosis induction [38]. The studied solutions also induced an increase in the expression of the *CDKN1A* gene and a lack of changes in the expression of the *CDKN1B* gene in PC-3 cells. Proteins encoded by both genes take part in cell cycle arrest. This agrees with reports indicating the high expression of the *CDKN1B* gene in androgen-dependent prostate cancer cells and a lack of its significance in the case of androgen-independent cells, such as PC-3 cells [40]. Breast cancer cells MDA-MB-231 did not exhibit any changes in the expression level of these genes. MCF-10A normal cells also exhibited a statistically significant increase in the expression level of *DDIT3* and *CDKN1B* genes, and in the case of the pure CBD solution, *BBC3*. The lack of any other reaction to the studied solutions in the administered concentration (IC50 calculated for the corresponding cancer line) suggests that the observed gene expression changes are insufficient to induce apoptosis. A similar pattern was observed in the case of PNT2 cells, which increased the expression of *DDIT3* and *BBC3* genes but only when incubated with pure CBD solution. 

Our results suggest the possible involvement of ROS in the mechanism of response to CBD and CBD-rich extracts. The effect elicited by α-TOC was significant in certain experimental settings but completely absent in others, and its magnitude was variable. The observed effects do not form any consistent pattern; therefore, it is impossible to form any final conclusions in this regard, indicating a need for further mechanistic studies, involving different assays, e.g., direct assessment or intracellular ROS levels with probes such as diacetyldichlorofluorescein.

Despite the promising results of many studies, the knowledge of interactions between cannabinoids and other *Cannabis* metabolites is still limited. A problem still waiting to be solved is to gain an understanding of the relation between *Cannabis* chemotypes (specifically exhibiting different THC/CBD ratio) and the response of the treated cells or organism. Therefore, it is necessary to conduct more preclinical studies focused on *Cannabis* metabolites interactions from the perspective of biochemistry and pharmacology, as well as clinical trials to assess the efficiency and safety of the optimal combinations of chemicals. 

Data regarding the anti-cancer potential of cannabinoids are mostly limited to preclinical studies conducted on cell lines and animal models. The first experiment conducted on human subjects was a small pilot study on nine patients with terminal-stage recurrent glioblastoma resistant to the standard therapy [41]. THC was administered intratumorally. This approach was safe, and patients did not exhibit any psychoactive effects. Certain patients exhibited a decrease in tumor growth. Changes observed upon THC administration in two patients could be connected with the anti-cancer effect of THC (decreased cell proliferation, the occurrence of apoptosis) [41]. Despite these promising observations, the obvious study limitations, such as small group size, hinder the possibility of drawing meaningful conclusions. This points to the need for further clinical trials that could help to assess the dosage and the potential interaction of cannabinoids with other substances.

Some ongoing or recently finished trials aim to assess the safety and the impact of cannabinoids or cannabinoid-based preparations on cancer patients. Examples include a study on the safety of nabiximols in combination with temozolomide in patients with recurrent glioblastoma [42,43]. The aim of another trial is to evaluate the impact of CBD as a single treatment in patients with solid tumors [44]. There are also studies assessing the safety and effects of dexanabinol (synthetic cannabinoid) in patients with solid tumors and brain cancer, and in healthy subjects [45,46,47].

An interesting idea is to combine cannabinoids with conventional anti-cancer drugs. The promising results of research on animal models of glioblastoma treated simultaneously with temozolomide and THC led to the aforementioned clinical trials [48,49,50]. Similar results were obtained in a study on a combination of gemcitabine with cannabinoids administered to pancreatic cancer cells [51]. Initial research on the coadministration of cannabinoids with radiotherapy also seems promising [52]. The main benefit of this approach is the possibility to target cancer progression on many levels while simultaneously reducing the toxicity of the agents used. 

We wish to stress several limitations of our study. Firstly, the research model was based on in vitro experiments models, which are important to explore the complexity of action but do not fully reflect the interactions and influences of other cell types, extracellular matrix components, and immune responses present in vivo. Secondly, our investigation was based on a limited number of cell lines, and, therefore, it does not fully capture the considerable cancer heterogeneity at the cellular and molecular levels. This can limit the generalizability of the findings and the ability to predict the response of heterogeneous tumors to cannabinoid treatments. Thirdly, the study involved the short-term exposure of cancer cells to cannabinoids, which does not reflect prolonged exposure in clinical settings. Another factor limiting the conclusions is that the concentrations used here may not be achievable in vivo or may lead to toxic effects. Last but not least, determining the physiologically clinically relevant doses of cannabinoids through in vitro studies is challenging. The elucidation of these issues requires further preclinical testing using animal models to explore the optimal administration routes, dosage, drug interactions, and safety profile.

## 4. Materials and Methods

*Chemicals.* We obtained a cannabidiol solution from Sigma Aldrich (90899, 10 mg/mL, Darmstadt, Germany). The *C. sativa* extracts were gifts from The Institute of Natural Fibres and Medicinal Plants (Poznań, Poland). The solutions were dissolved in ethanol. The extraction protocol and extract composition have been described previously [53]. 

*Cell culture.* The human breast cancer cell line (MDA-MB-231, HTB-26™), human prostate cancer line (PC-3, CRL-1435™), human mammary gland cell line (MCF-10A, CRL-10317™), and human embryonic kidney cell line (HEK-293, CRL-1573™) were purchased from the American Type Culture Collection (ATCC^®^, Manassas, VA, USA). The human prostate cell line (PNT2, 95012613) was purchased from Sigma-Aldrich. The human fibroblast cell line (MSU-1.1) was a kind gift from NanoBioMedical Centre, Adam Mickiewicz University in Poznań. Cells were grown in a 1:1 mixture of Dulbecco’s modified Eagle’s medium (D5796, Sigma-Aldrich, Darmstadt, Germany) and nutrient mixture F-12 (N6658, Sigma-Aldrich), supplemented with 10% (*v/v*) fetal bovine serum (F4135, Sigma-Aldrich) and 1% (*v/v*) penicillin/streptomycin (P4333, Sigma-Aldrich). Cells were maintained at 37 °C in a humidified atmosphere of 5% CO_2_. In order to conduct cell passage, the cells were rinsed with Hank’s Balanced Salt Solution (H9269, Sigma Aldrich) to remove residual FBS. Subsequently, the cells were incubated with 750 μL of 0.25% trypsin-EDTA solution for 5 min at 37 °C. The detachment of cells from the vessel surface and their dispersion were assessed using an inverted microscope (Axiovert 200, Zeiss, Oberkochen, BW, Germany). After detachment, cells were resuspended in 8 mL of complete growth medium and divided into two new vessels. The cells were subjected to no more than six passages in culture when used in experiments. All the experiments described below were performed in triplicates. 

*Morphological changes assessment.* Cells were cultured in 25 cm^2^ flasks until they reached a confluence of approximately 80%. Then, media were replaced with standard or low-serum (0.5% FBS) media, containing appropriate concentrations of CBD, *C. sativa* extracts, or vehicle. After 24 h, cell images were captured by ZOE^TM^ Fluorescent Cell Imager (Bio-Rad, Hercules, CA, USA).

*Cell viability assay.* Cells were seeded in 96-well microplates at 5 × 10^3^ cells/well and cultured in an incubator for 24 h. Then, media were replaced with standard or low-serum (0.5% FBS) media containing appropriate concentrations of CBD, *C. sativa* extracts, or vehicle. After 24 h, Cell Counting Kit-8 assay (96992, Sigma Aldrich) was used according to the manufacturer’s instructions. The kit utilizes the reduction reaction of tetrazolium salt WST-8 to formazan by cellular dehydrogenases. The resulting formazan quantity is proportional to the number of metabolically active (living) cells in the population. Absorbance readings were taken at 450 nm using the ELx808 microplate reader (BioTek, Winooski, VT, USA). To calculate the half maximal inhibitory concentration (IC50), the online tool IC50 Calculator (AAT Bioquest, https://www.aatbio.com/tools/ic50-calculator (accessed on 28 January 2019)) was used. It determines the IC50 parameter by using a four-parameter logistic regression model.

*ROS involvement assay.* Cells were prepared as described above. Then, α-tocopherol (α-TOC) was added to the final concentration of 10 µM. α-TOC is a potent antioxidant. If reactive oxygen species (ROS) are a key component of the pathway leading to apoptosis, adding α-TOC should neutralize or reduce the effect of their activity. Cell viability was assessed by Cell Counting Kit-8 assay according to the manufacturer’s instructions.

*Apoptotic activity—luminometric assay.* Cells were seeded in 96-well microplates at 5 × 10^3^ cells/well and cultured in an incubator for 24 h. Then, media were replaced with standard or low-serum (0.5% FBS) media containing appropriate concentrations of CBD, *C. sativa* extracts, or vehicle. After 24 h, Caspase-Glo^®^ 3/7 Assay (PROMEGA, Madison, VI, USA) was used according to the manufacturer’s instructions. The reaction involves the lysis of cells and the release of their contents into the medium upon adding the kit’s reaction reagent to the cell culture. The reaction reagent contains a substrate with a tetrapeptide sequence DEVD, cleaved proteolytically due to the activity of caspases 3/7, resulting in the generation of aminoluciferin. This substance acts as a substrate for luciferase present in the reaction reagent, leading to a stable ‘glow-type’ luminescent signal. The luminescence intensity is proportional to the activity of caspases 3/7 in the reaction mixture.

*Apoptotic activity—Muse™ Caspase-3/7 Kit.* Cells were seeded in 6-well plates at a concentration of 1.5 × 10^5^ cells/well and cultured in an incubator for 24 h. Then, media were replaced with standard or low-serum (0.5% FBS) media, containing appropriate concentrations of CBD, *C. sativa* extracts, or vehicle. After 24 h, Muse™ Caspase-3/7 Kit (LUMIMCH100108, Merck, Darmstadt, Germany) was used according to the manufacturer’s instructions. Cells were analyzed using Muse™ Cell Analyzer (Merck, Darmstadt, Germany). The kit includes NucView™ reagent containing a dye binding to DNA linked with the peptide substrate DEVD. The dye, initially unable to bind DNA, is released upon proteolytic cleavage of DEVD due to caspases 3/7 activity, resulting in a fluorescent signal. The signal intensity is proportional to the activity of caspases 3/7. Additionally, the kit contains the cell death marker 7-AAD, a fluorescent dye penetrating dead cells through damaged cell membranes and binding to their nucleic acids.

*Apoptotic activity—flow cytometry analysis.* Cells were seeded in 6-well plates at a concentration of 1.5 × 10^5^ cells/well and cultured in an incubator for 24 h. Then, media were replaced with standard or low-serum (0.5% FBS) media, containing appropriate concentrations of CBD, *C. sativa* extracts, or vehicle. After 24 h, FITC Annexin V Apoptosis Detection Kit with PI (BioLegend, San Diego, CA, USA) was used according to the manufacturer’s instructions. Cells were analyzed by flow cytometry using CyFlow^®^ Cube 8 (Sysmex, Warsaw, Poland). The assay utilizes changes in the permeability of the cell membrane for propidium iodide and the translocation of phosphatidylserine (PS) across the cell membrane. In normal cells, PS is present in the intracellular leaflet of the plasma membrane, but during early apoptosis, membrane asymmetry is lost, and PS is translocated to the external leaflet. The kit employed high-affinity annexin V (human anticoagulant) labeled with fluorescein isothiocyanate (FITC) to bind the translocated PS, enabling the identification of apoptotic cells. Additionally, propidium iodide (PI), a fluorescent dye, entered dead cells through damaged cell membranes, binding to their nucleic acids and facilitating their detection.

*RNA Isolation and Reverse Transcription-Polymerase Chain Reaction*. Cells were cultured in 25 cm^2^ flasks until they reached the confluence of approximately 80%. Then, media were replaced with low-serum (0.5% FBS) media, containing vehicle or CBD and *C. sativa* extracts at concentrations equal to their calculated IC50. After 24 h, total RNA was isolated from cells using the Absolutely RNA Miniprep Kit (Agilent, Santa Clara, CA, USA) according to the manufacturer’s instructions. The RNA concentration was measured using a UV-Vis spectrophotometer (NanoDrop 2000, Thermo Scientific, Waltham, MA, USA). The RNA integrity was assessed by 1.5% agarose gel electrophoresis. A total of 500 ng of RNA was reverse transcribed at 46 °C using iScript™ cDNA Synthesis Kit (Bio-Rad, Hercules, CA, USA). The quality of the reverse transcription reaction was assessed by PCR amplification of the GAPDH gene and the subsequent 1.5% agarose gel electrophoresis (sequences of the primers used are listed in Supplementary Table S1). The quantitative polymerase chain reaction (qPCR) was performed using SsoAdvanced™ Universal SYBR^®^ Green Supermix (Bio-Rad, Hercules, CA, USA) and primers in the form of PrimePCR™ SYBR^®^ Green Assay (Bio-Rad, Hercules, CA, USA) for the following genes: *ACTB*, *GAPDH*, *BBC3*, *CDKN1A*, *CDKN1B*, *DDIT3*, and *PTGS2*. The amplification was performed on the CFX96 Touch™ Real-Time PCR Detection System (BIO-RAD, Hercules, CA, USA) as follows: initial denaturation at 95 °C for 2 min, 40 cycles consisting of denaturation at 95 °C for 5 s, and annealing/synthesis at 60 °C for 30 s. The melt curve protocol was subsequently performed in 5 s increments at 0.5 °C from 65 °C to 95 °C. Data preprocessing and normalization were performed using BIO-RAD CFX Manager (BIO-RAD, Hercules, CA, USA).

*Statistical analysis.* The results are presented as the mean ± SD. The results were positively tested for normal distribution (Shapiro test) and homogeneity of variance (Bartlett test). For comparison between mean values, the ANOVA test was used, with Tukey’s HSD test employed for post hoc analysis for data with homogeneity of variance. For data without homogeneity of variance, the Dunnett T3 test was used. Values of *p* < 0.05 were considered significant. All of the statistical analyses were performed using R version 3.4.1.

## Figures and Tables

**Table 1:** Main cannabinoid content in *C. sativa* extracts used in the study. CBD, cannabidiol; CBDA, cannabidiolic acid; ∆9THC, ∆9-tetrahydrocannabinol; and ∆9THCA, ∆9-tetrahydrocannabinolic acid.

Extract	Component (mg/g)			
	CBD	CBDA	∆9THC	∆9THCA
B	215.2	1.2	13.3	0.15
D	220.2	1.2	15.5	0.1

**Table 2:** Calculated IC50 concentration of the studied solutions. Asterisk—statistically significant difference between values obtained for a cancer cell line and its corresponding normal counterpart: **—*p* < 0.01; and *—*p* < 0.05. Hash—statistically significant difference between values obtained for corresponding treatments in the standard medium conditions and low-FBS medium conditions: ###—*p* < 0.001; and #—*p* < 0.05. The IC50 value for certain experimental settings was impossible to accurately calculate since it exceeded the studied concentration range (0–15 µM); such cases were denoted as >15 µM.

Cell Line	Standard Medium (10% FBS)			Low-FBS Medium (0.5% FBS)		
	CBD	Extract B	Extract D	CBD	Extract B	Extract D
MDA-MB-231	12.26 µM	9.31 µM	10.76 µM	6.04 µM * ###	6.01 µM **	6.09 µM #
PC-3	12.04 µM	12.97 µM	12.2 µM	8.14 µM	11.58 µM	10.98 µM
MSU-1.1	>15 µM	>15 µM	>15 µM	5.51 µM	6.28 µM	6.29 µM
HEK-293	13.45 µM	12.06	12.02 µM	5.43 µM ###	6.49 µM ###	7.3 µM ###
MCF-10A	>15 µM	>15 µM	>15 µM	10.27 µM	12.91 µM	>15 µM
PNT2	>15 µM	>15 µM	>15 µM	11.9 µM	>15 µM	>15 µM

## Data Availability

Data are contained within the article and Supplementary Materials.

## Supplementary Materials

The following supporting information can be downloaded at: https://www.mdpi.com/article/10.3390/molecules28237887/s1, Figures S1–S12. Representative images of the cells used in the study after 24 h of incubation with studied solutions in either standard (left panels) or low-FBS (right panels) medium conditions. Table S1. Primers used in the quality assessment of the reverse transcription.
