# Supplementary material for: In Vitro Evidence of Selective Pro-Apoptotic Action of the Pure Cannabidiol and Cannabidiol-Rich Extract
Source: Molecules. 2023 Dec 1;28(23):7887. doi: 10.3390/molecules28237887 (PMC10708261; doi:10.3390/molecules28237887)
Supplement: Supplementary file 1 [file molecules-28-07887-s001.zip › Supplementary Figures captions.pdf]

Supplementary Figures S1–S12. Representative images of the cells used in the study after 24 hours of incubation with studied solutions in either standard (left panels) or low-FBS (right panel) medum conditions.

Supplementary Figure S1. MDA-MB-231 cells incubated with CBD solution.

Supplementary Figure S2. MDA-MB-231 cells incubated with extract B solution.

Supplementary Figure S3. MDA-MB-231 cells incubated with extract D solution.

Supplementary Figure S4. PC-3 cells incubated with CBD solution.

Supplementary Figure S5. PC-3 cells incubated with extract B solution.

Supplementary Figure S6. PC-3 cells incubated with extract D solution.

Supplementary Figure S7. PNT2 cells incubated with CBD solution.

Supplementary Figure S8. PNT2 cells incubated with extract B solution.

Supplementary Figure S9. PNT2 cells incubated with extract D solution.

Supplementary Figure S10. MCF-10A cells incubated with CBD solution.

Supplementary Figure S11. MCF-10A cells incubated with extract B solution.

Supplementary Figure S12. MCF-10A cells incubated with extract D solution.
