# Supplementary material for: In Vitro Evidence of Selective Pro-Apoptotic Action of the Pure Cannabidiol and Cannabidiol-Rich Extract
Source: Molecules. 2023 Dec 1;28(23):7887. doi: 10.3390/molecules28237887 (PMC10708261; doi:10.3390/molecules28237887)
Supplement: Supplementary file 1 [file molecules-28-07887-s001.zip › Supplementary Table S1.pdf]

Supplementary Table S1. Primers used in the quality assessment of the reverse transcription.

**Primer**	**Sequence**
GAPDH-F	5′-GGGCATGAAGAATGAGAAGT-3′
GAPDH-R	5′-GTCTTCTGGGTGGCAGTGAT-3′
