# Supplementary figures and images for: In Vitro Evidence of Selective Pro-Apoptotic Action of the Pure Cannabidiol and Cannabidiol-Rich Extract
Source: Molecules. 2023 Dec 1;28(23):7887. doi: 10.3390/molecules28237887 (PMC10708261; doi:10.3390/molecules28237887)

**10% FBS**

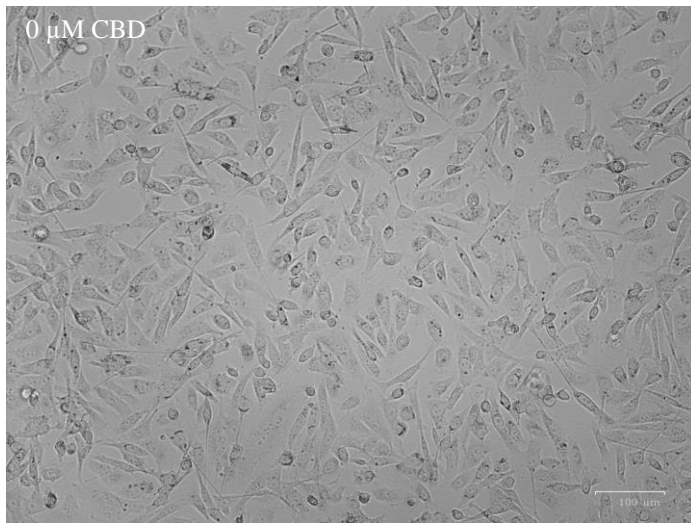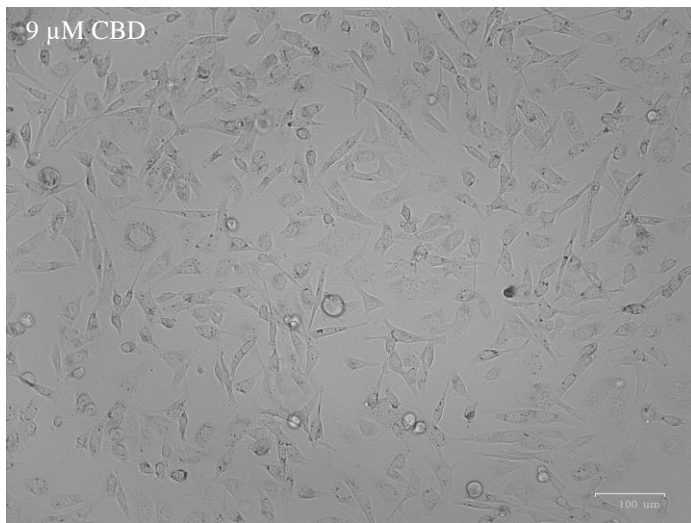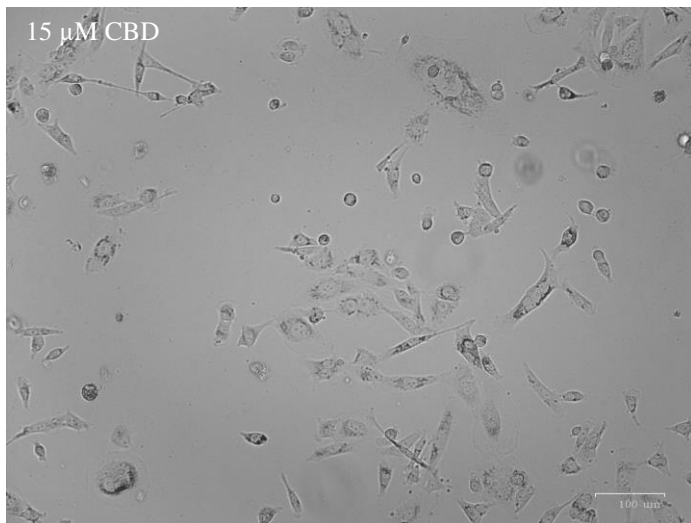

**0.5% FBS**

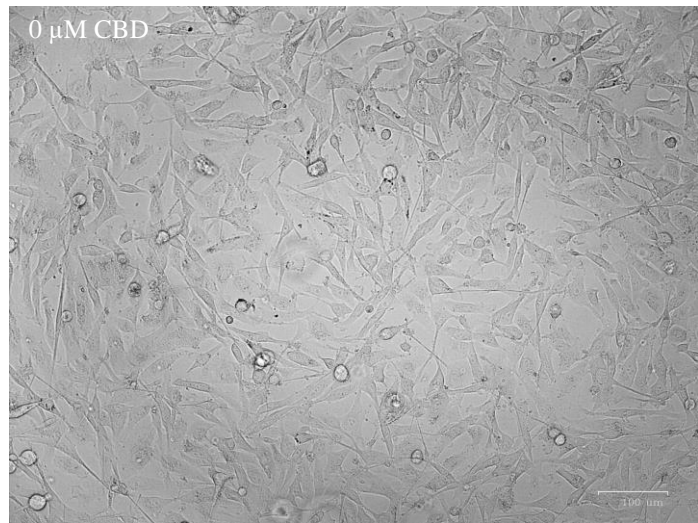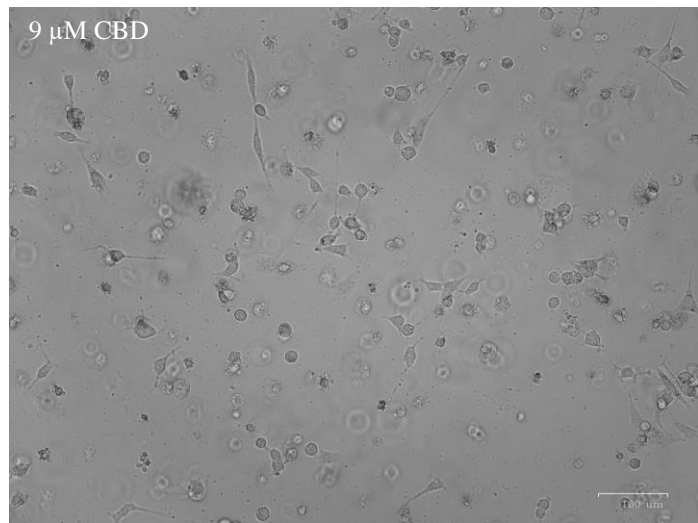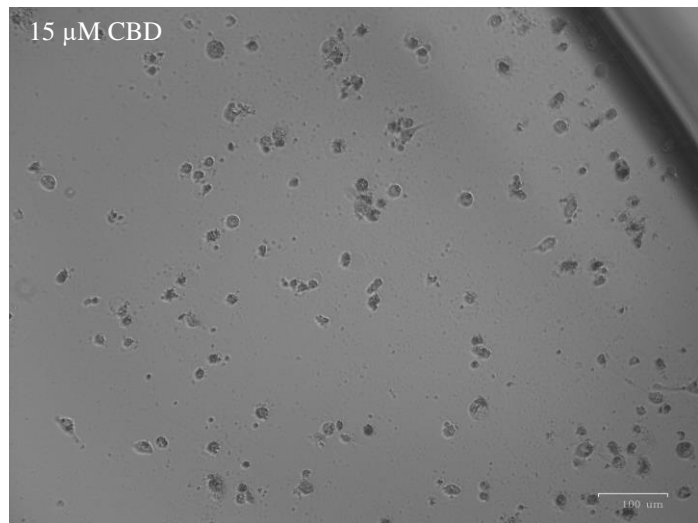

Supplement: Supplementary file 1 [file molecules-28-07887-s001.zip › Supplementary Figure S1 MDA CBD.pdf]

## 10% FBS

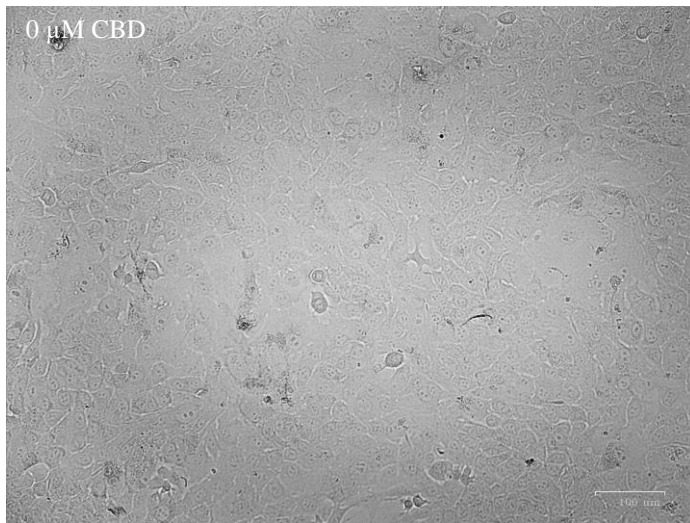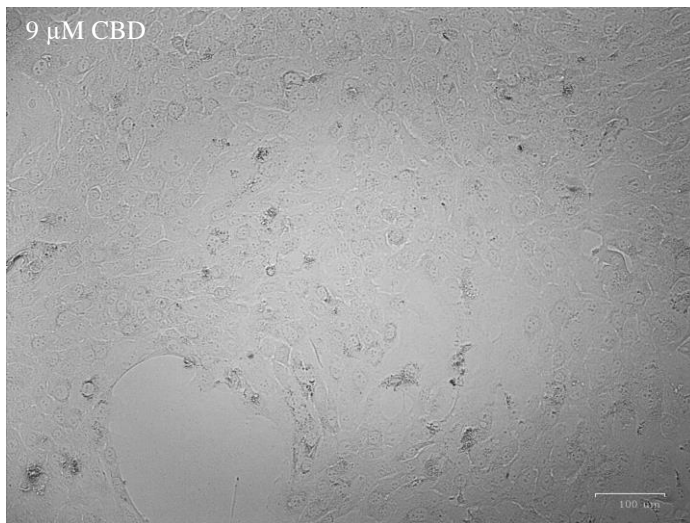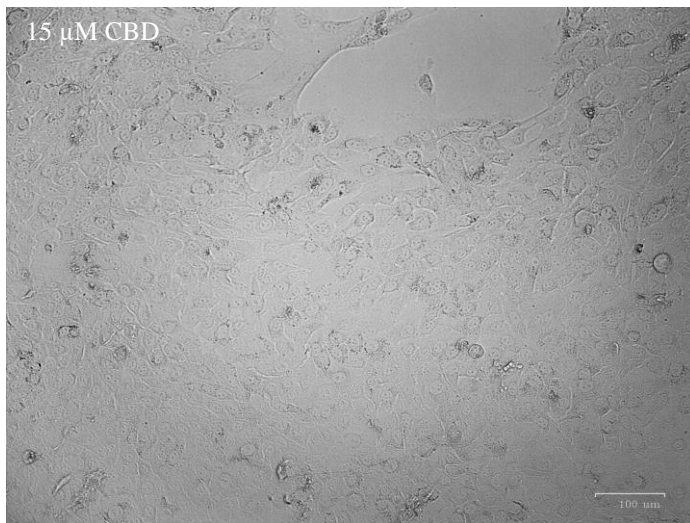

## 0.5% FBS

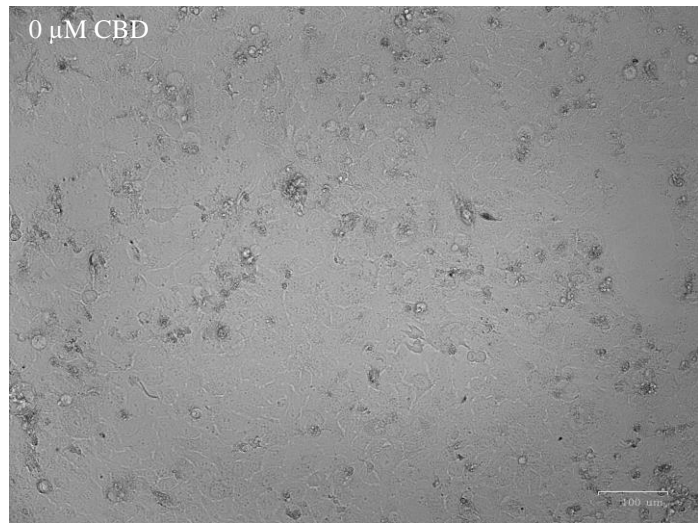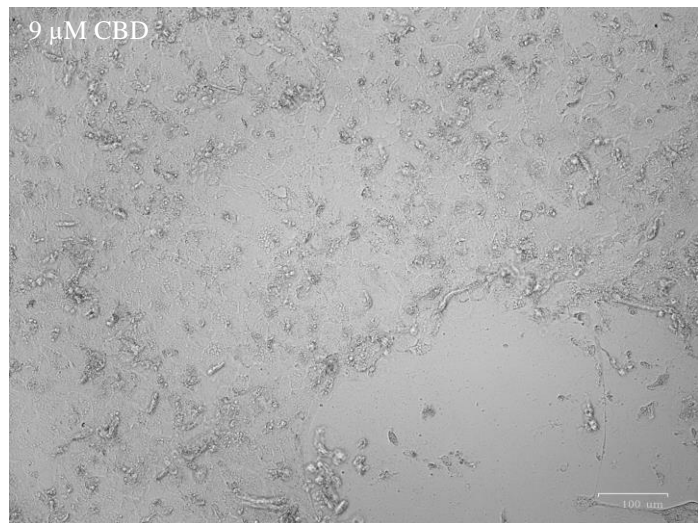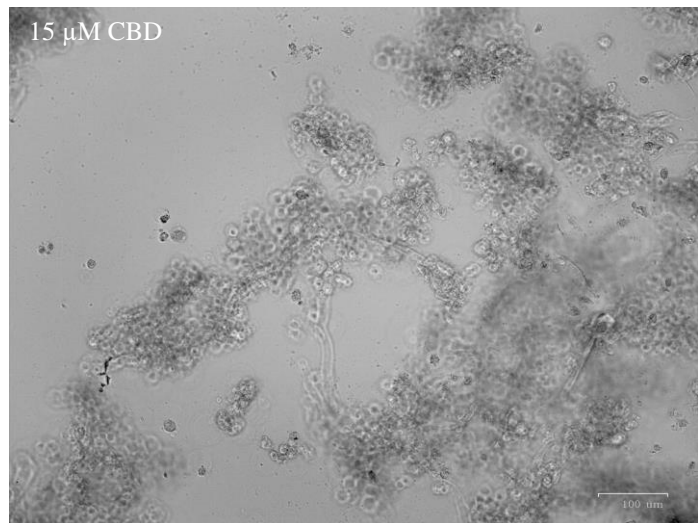

Supplement: Supplementary file 1 [file molecules-28-07887-s001.zip › Supplementary Figure S10 MCF10 CBD.pdf]

**10% FBS**

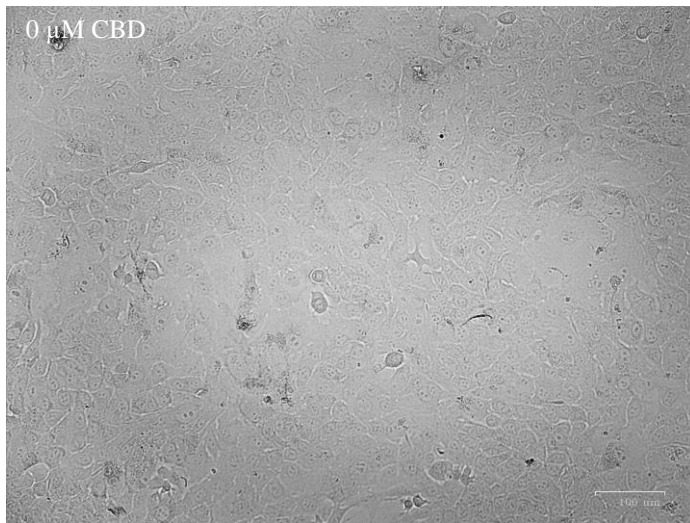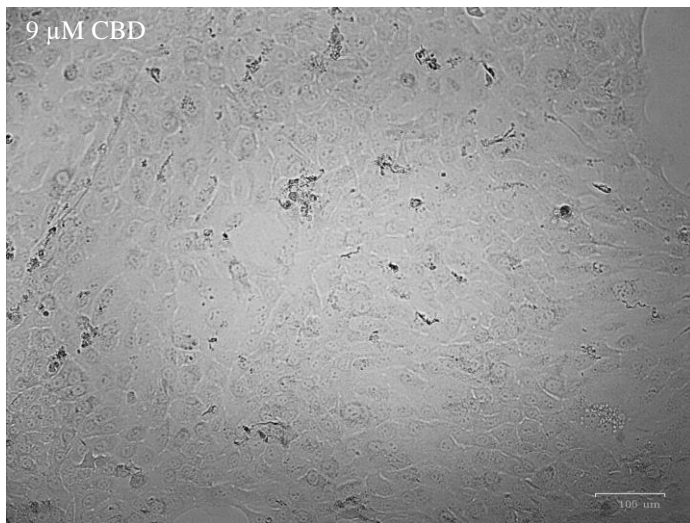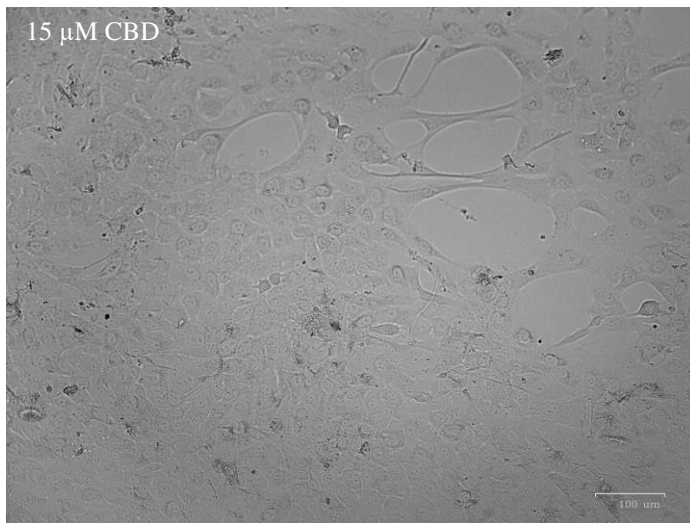

**0.5% FBS**

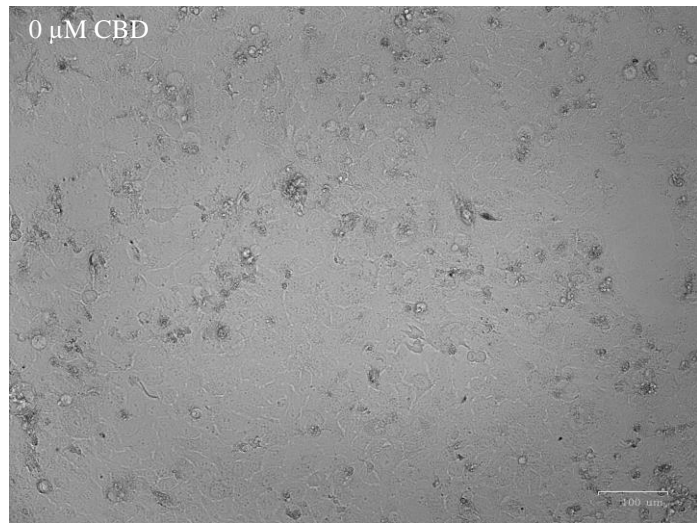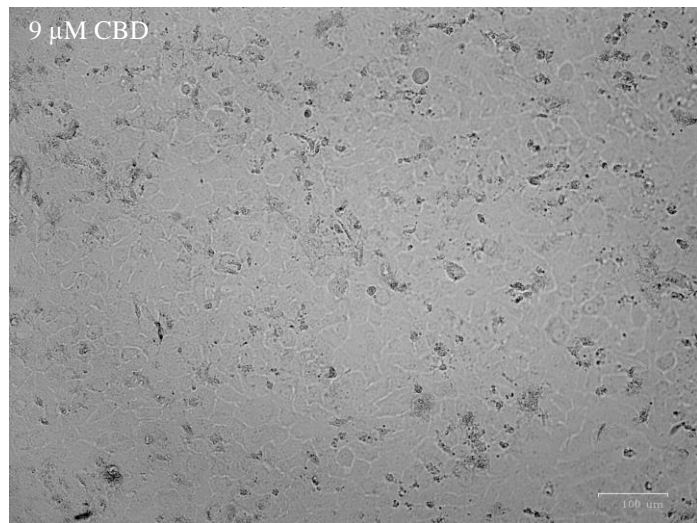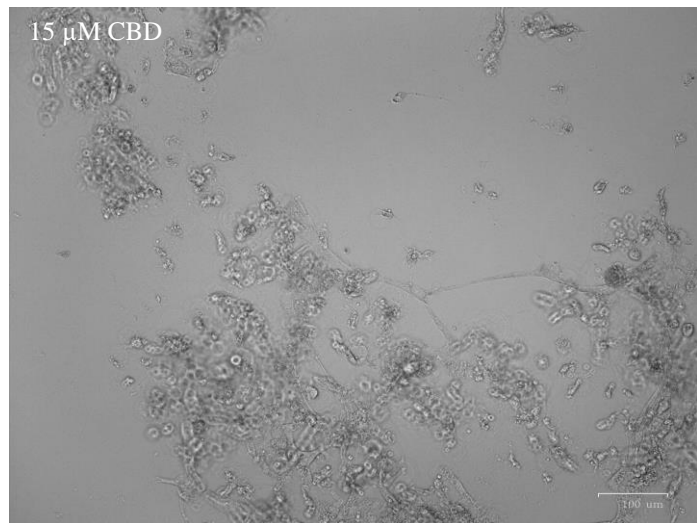

Supplement: Supplementary file 1 [file molecules-28-07887-s001.zip › Supplementary Figure S11 MCF10 EB.pdf]

## 10% FBS

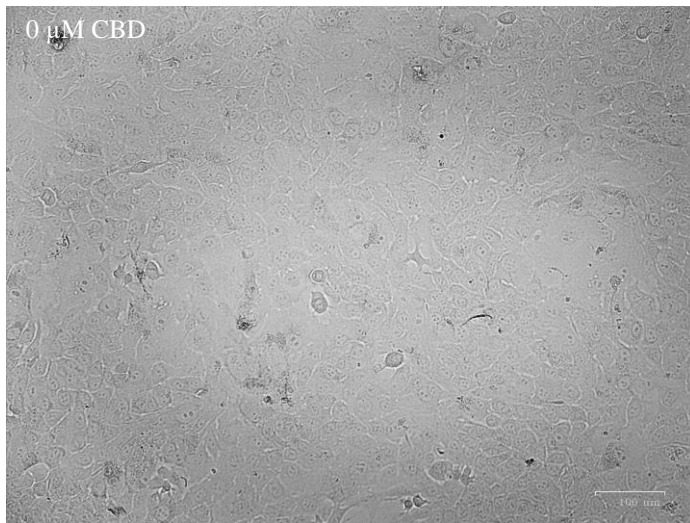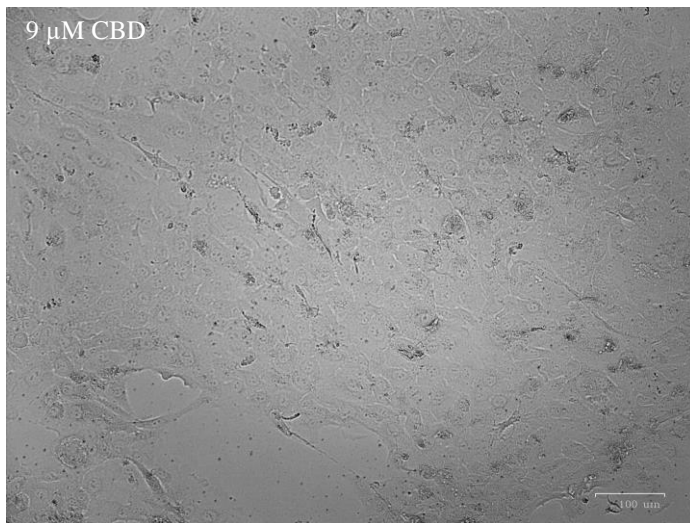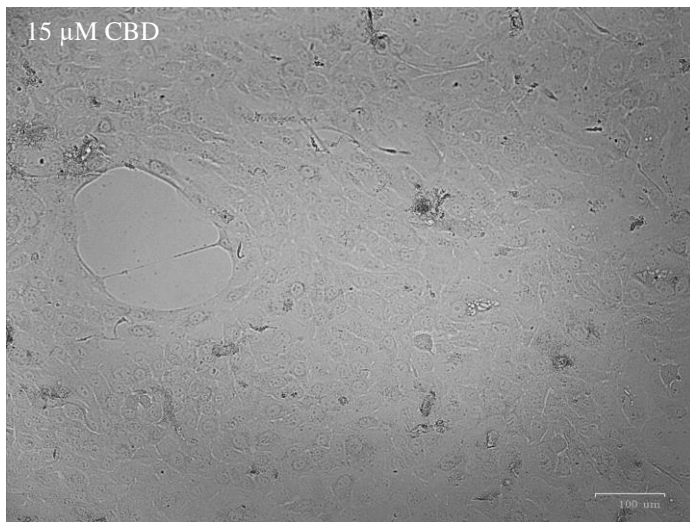

## 0.5% FBS

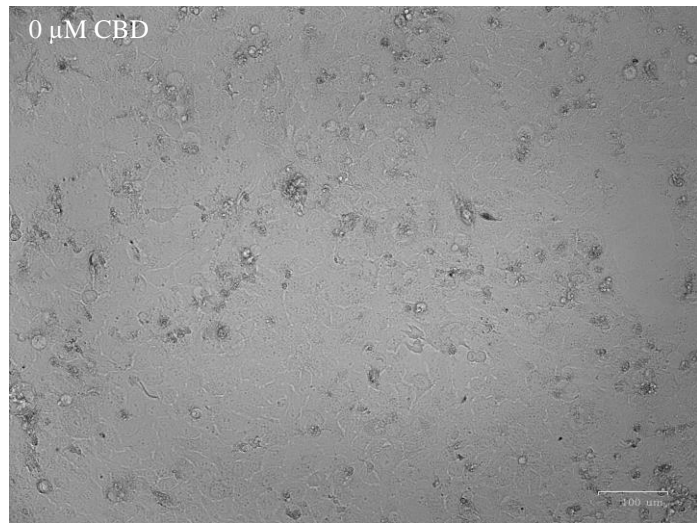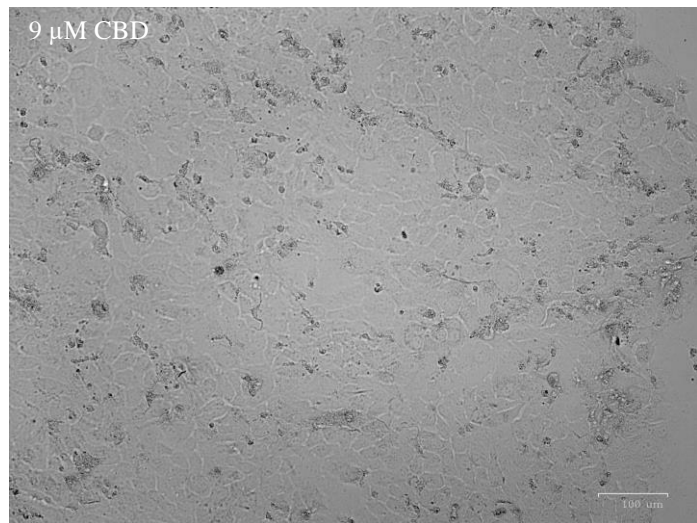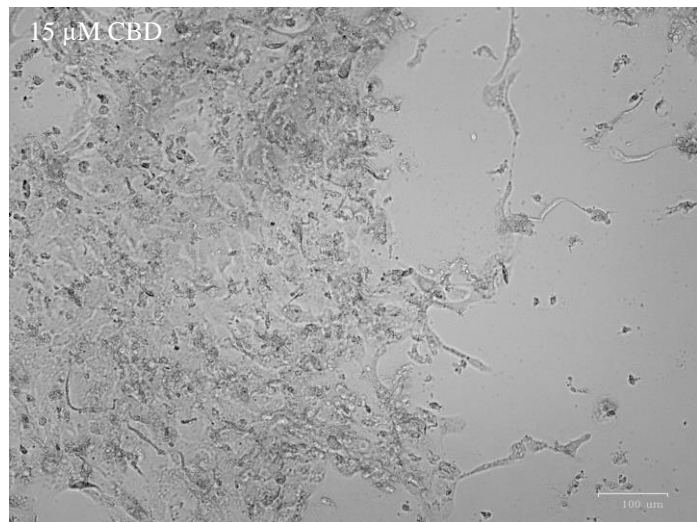

Supplement: Supplementary file 1 [file molecules-28-07887-s001.zip › Supplementary Figure S12 MCF10 ED.pdf]

**10% FBS**

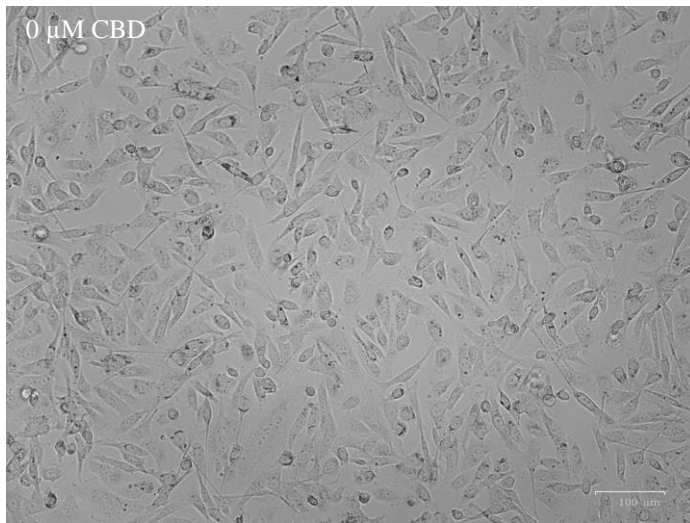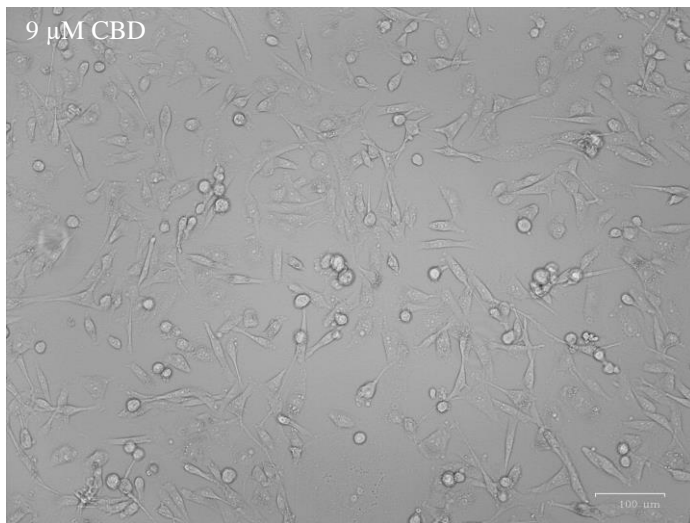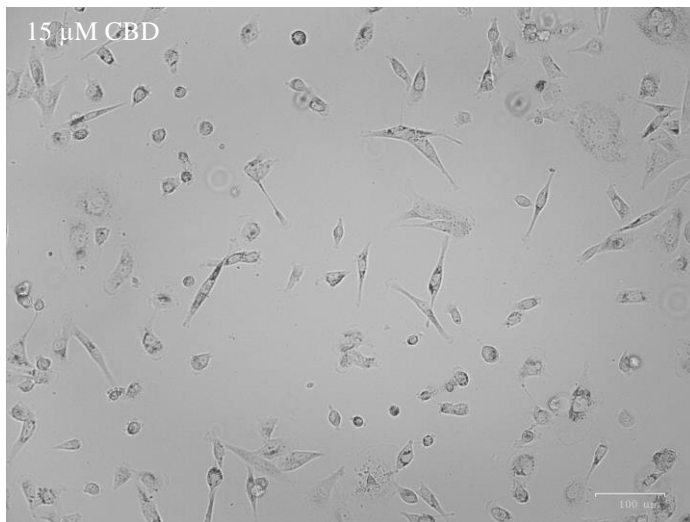

**0.5% FBS**

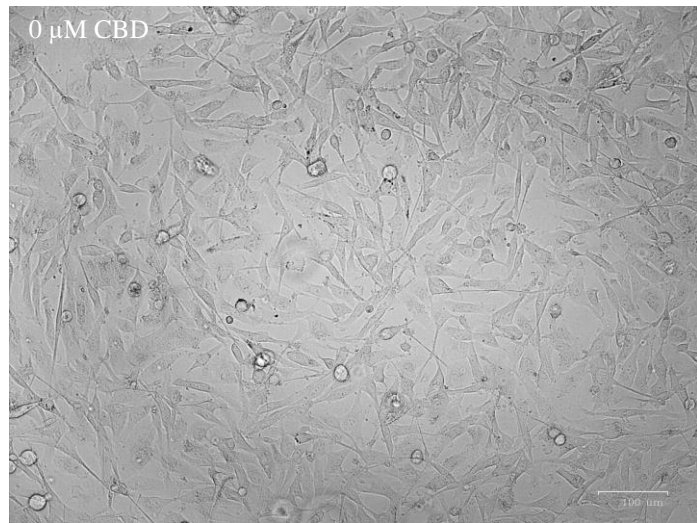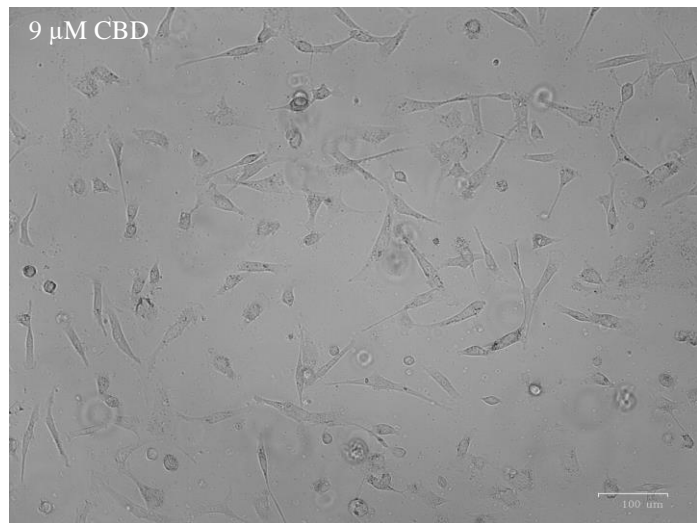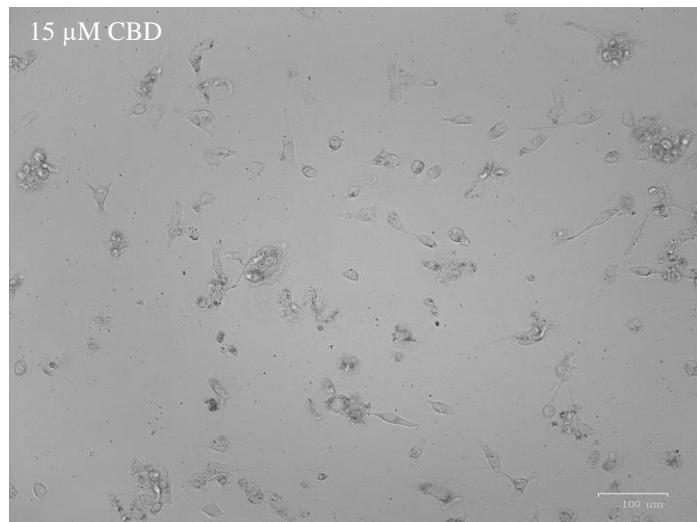

Supplement: Supplementary file 1 [file molecules-28-07887-s001.zip › Supplementary Figure S2 MDA EB.pdf]

**10% FBS**

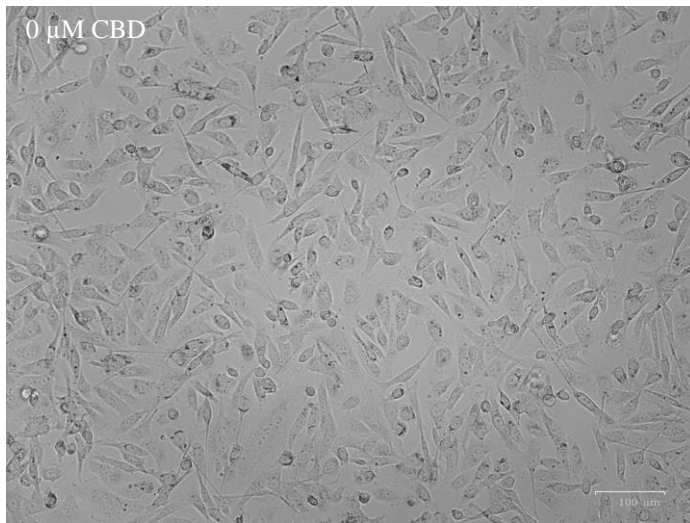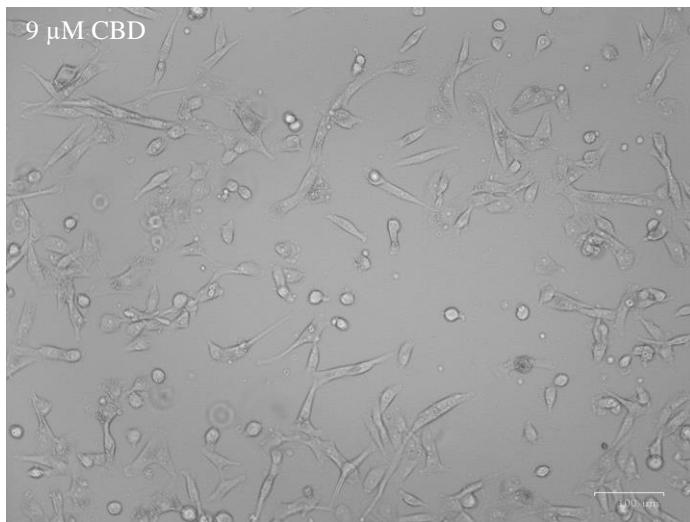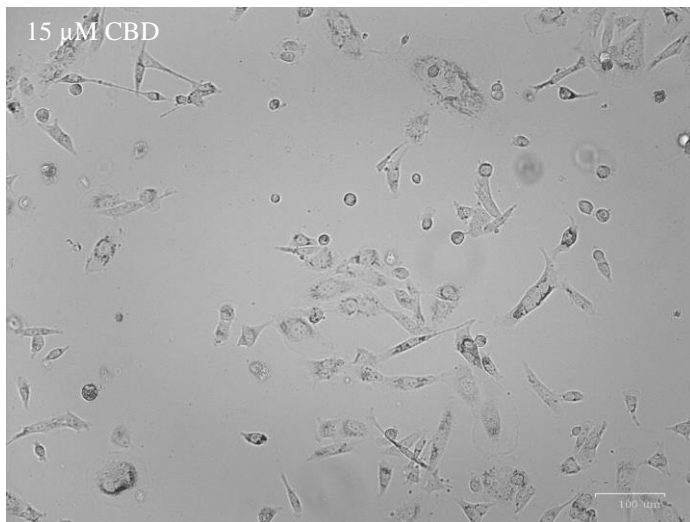

**0.5% FBS**

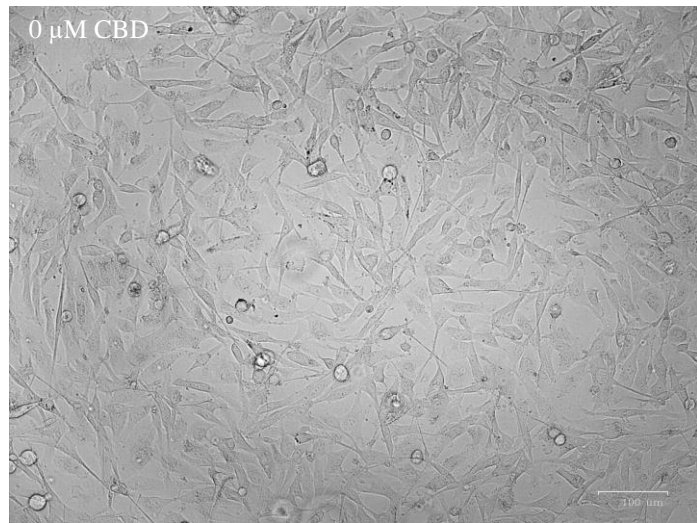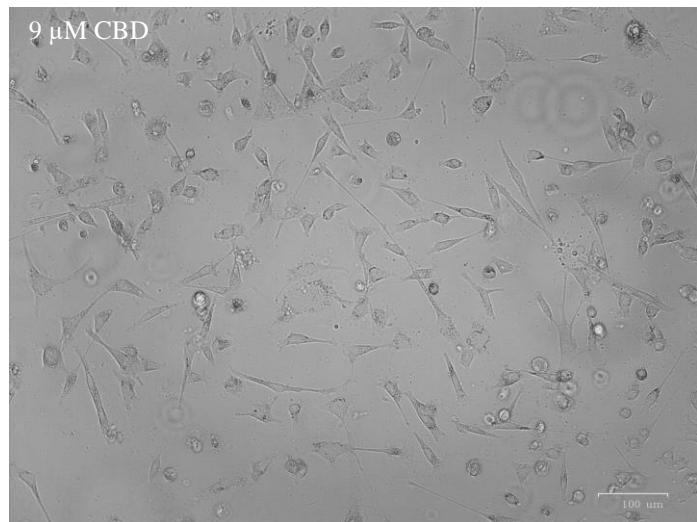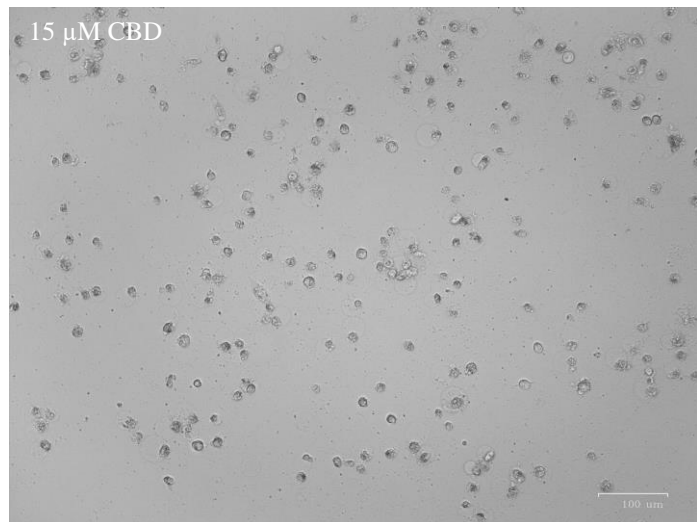

Supplement: Supplementary file 1 [file molecules-28-07887-s001.zip › Supplementary Figure S3 MDA ED.pdf]

**10% FBS**

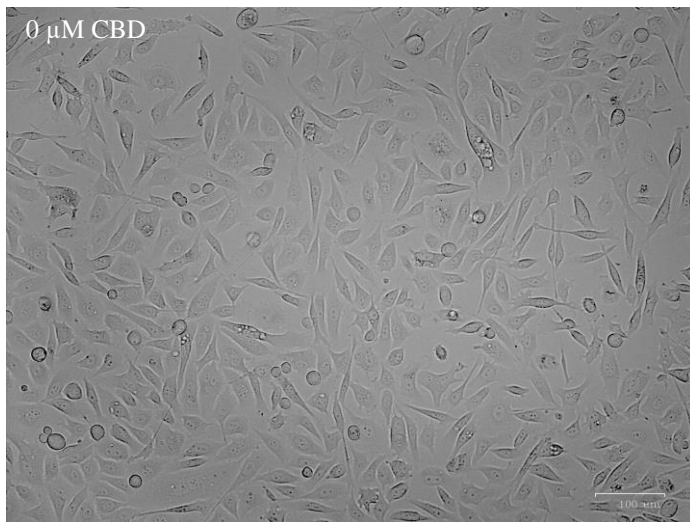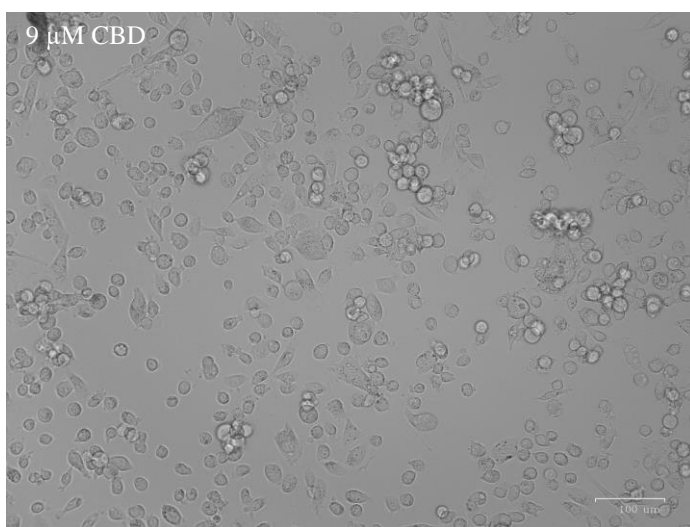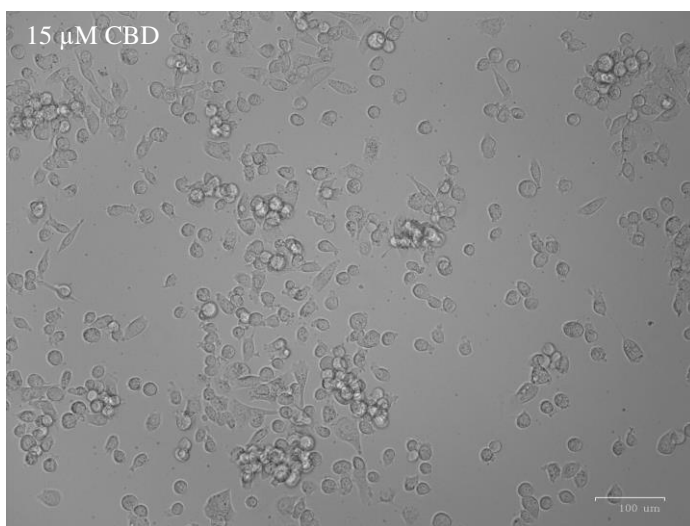

**0.5% FBS**

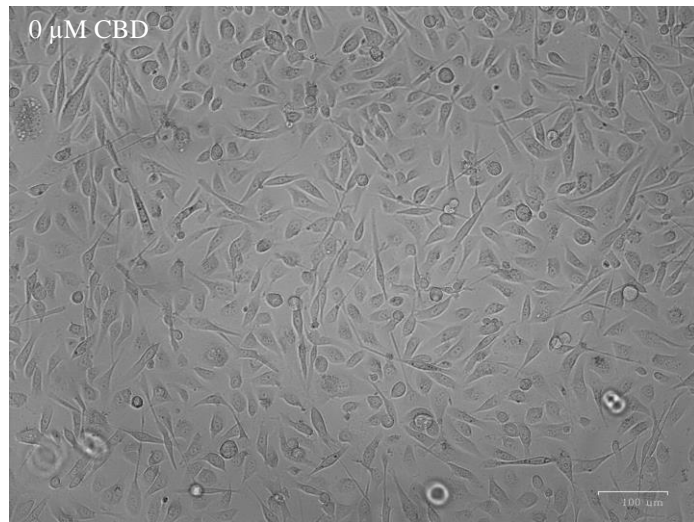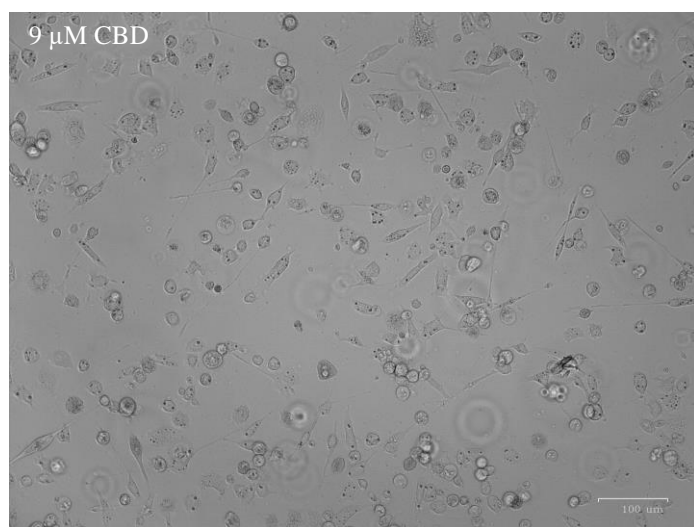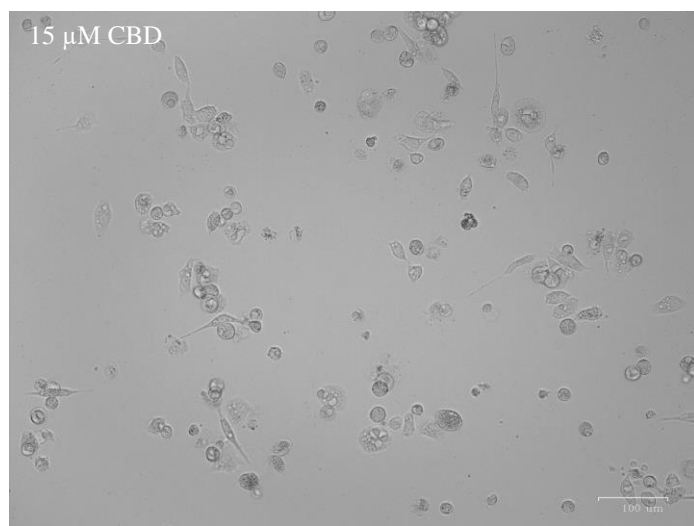

Supplement: Supplementary file 1 [file molecules-28-07887-s001.zip › Supplementary Figure S4 PC3 CBD.pdf]

**10% FBS**

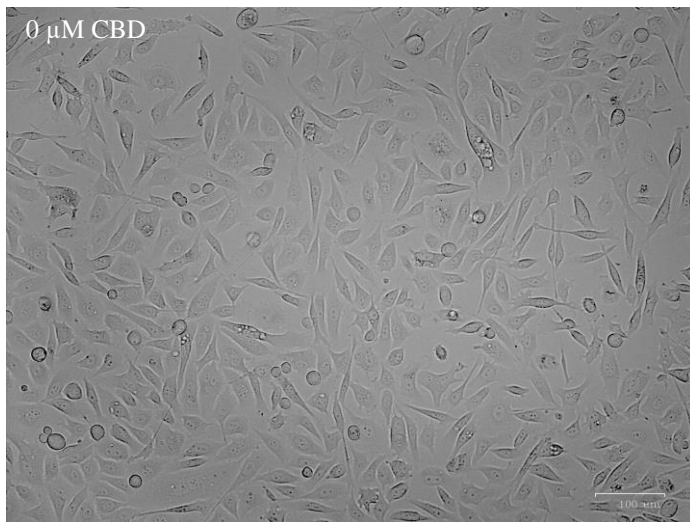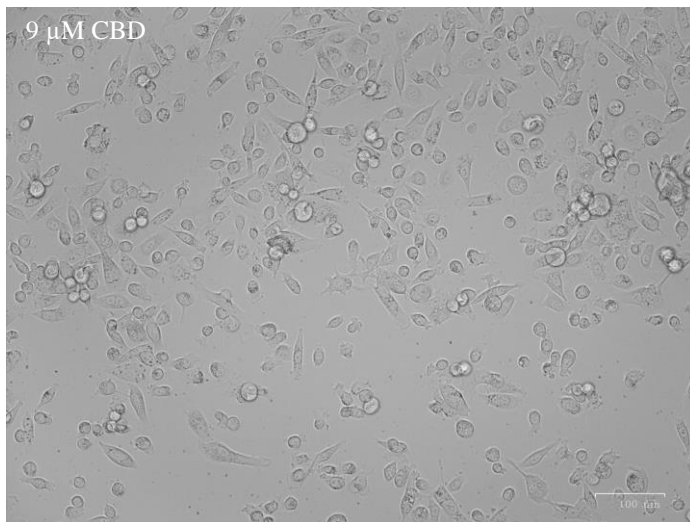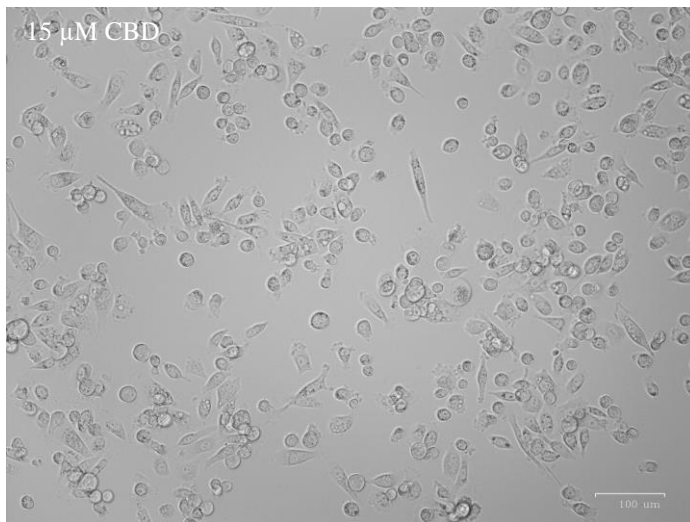

**0.5% FBS**

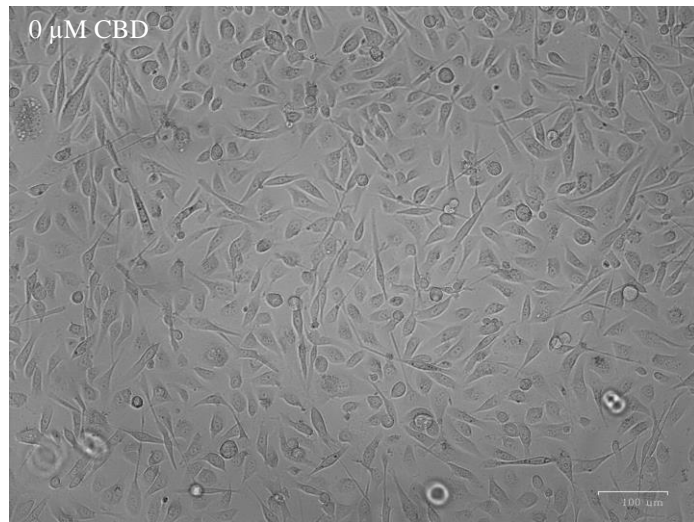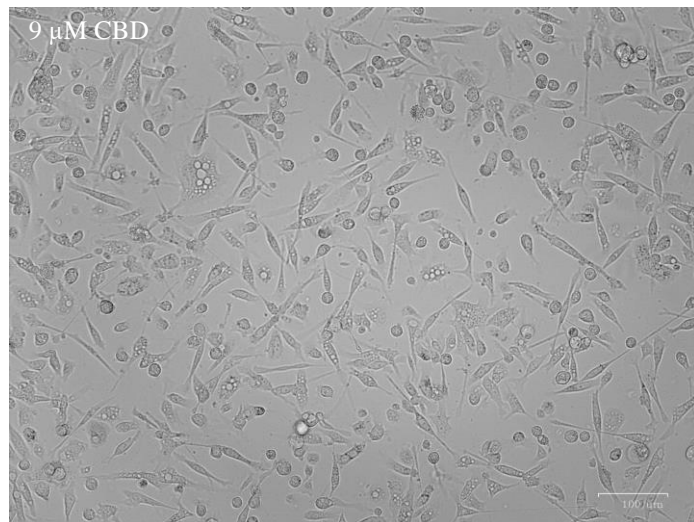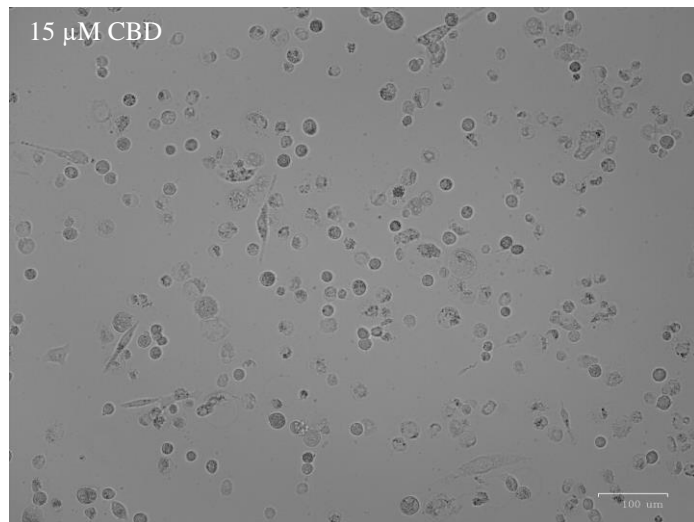

Supplement: Supplementary file 1 [file molecules-28-07887-s001.zip › Supplementary Figure S5 PC3 EB.pdf]

**10% FBS**

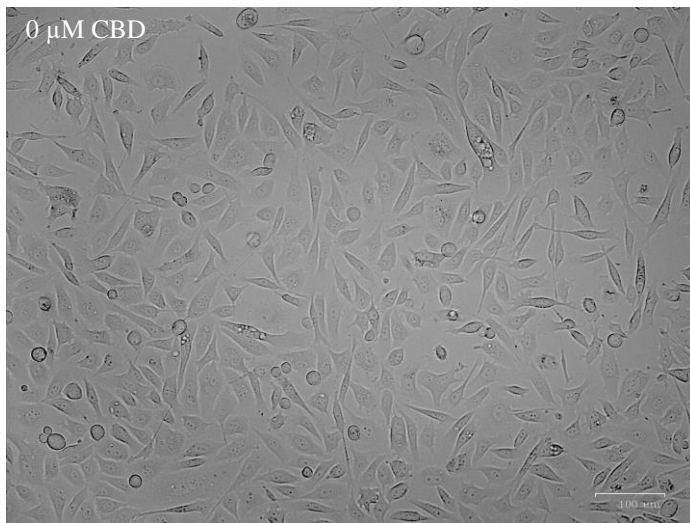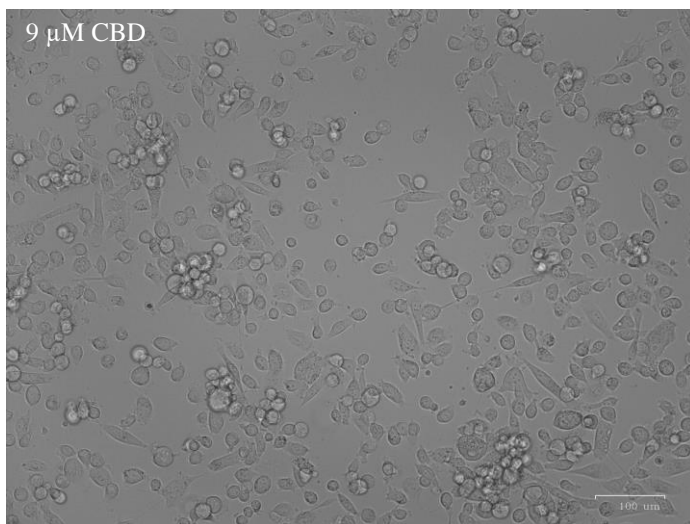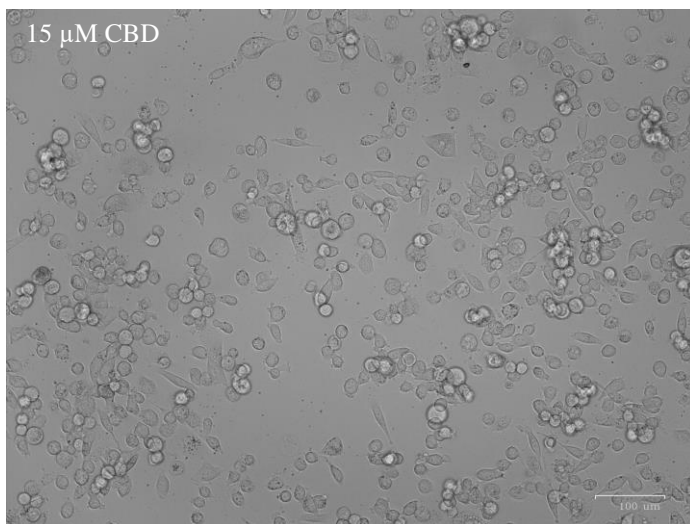

**0.5% FBS**

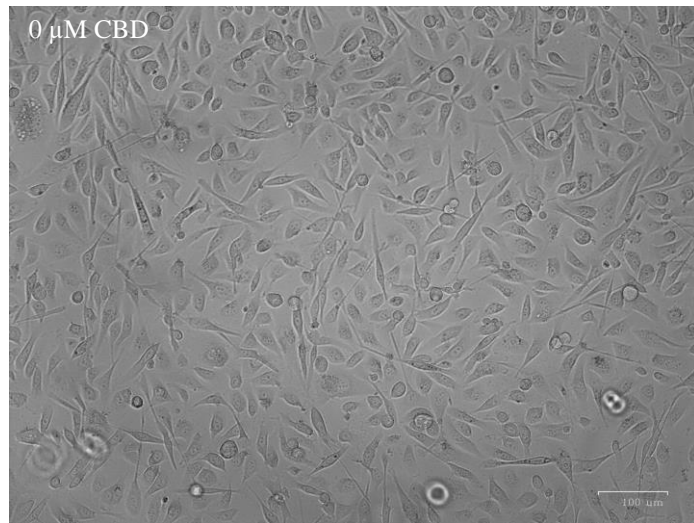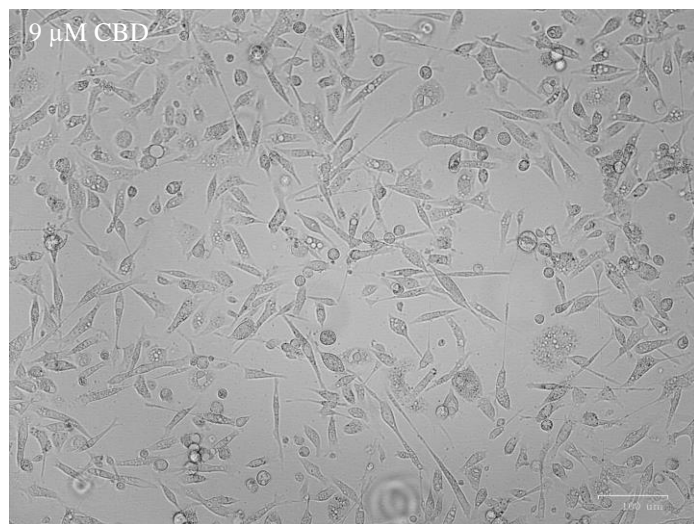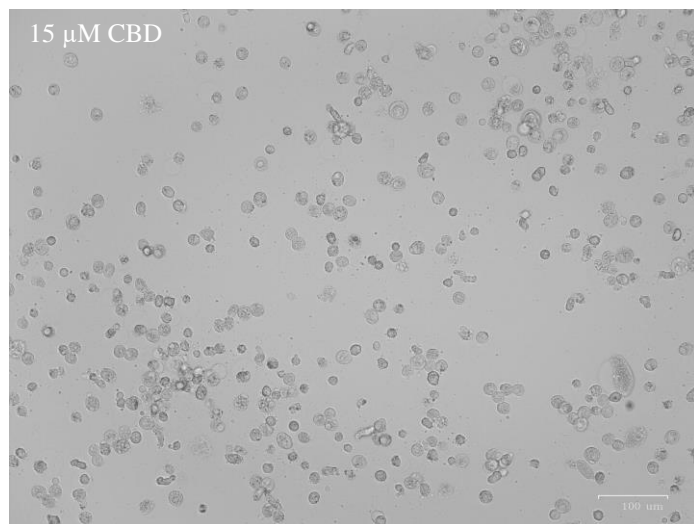

Supplement: Supplementary file 1 [file molecules-28-07887-s001.zip › Supplementary Figure S6 PC3 ED.pdf]

## 10% FBS

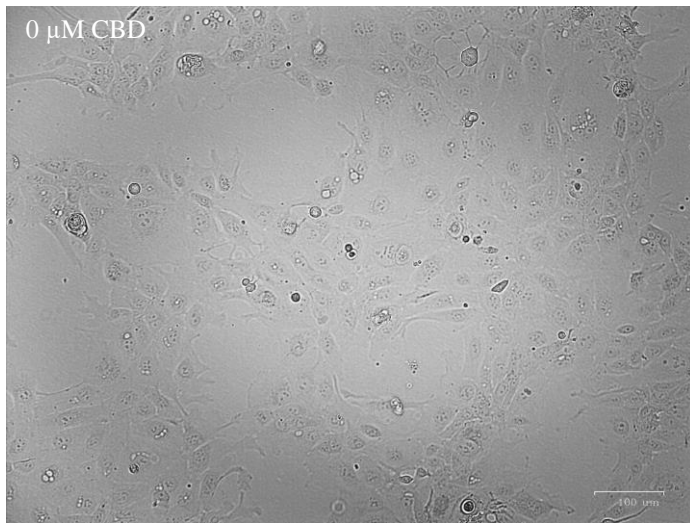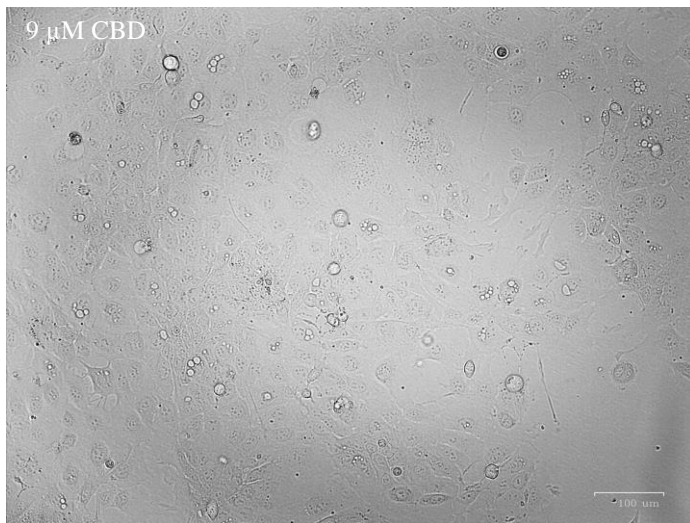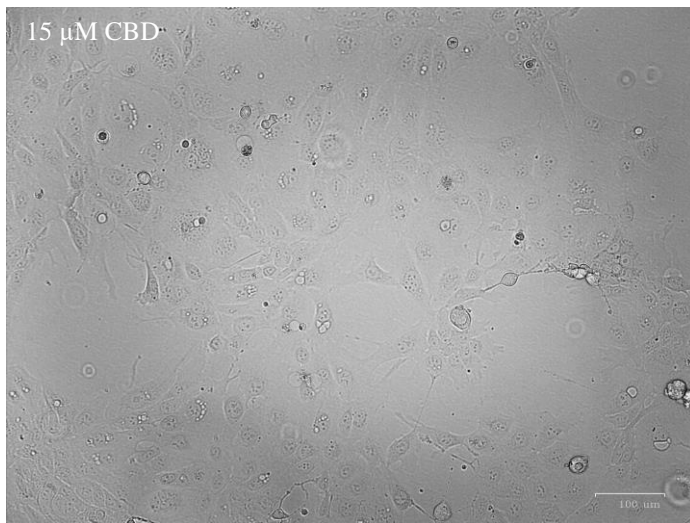

## 0.5% FBS

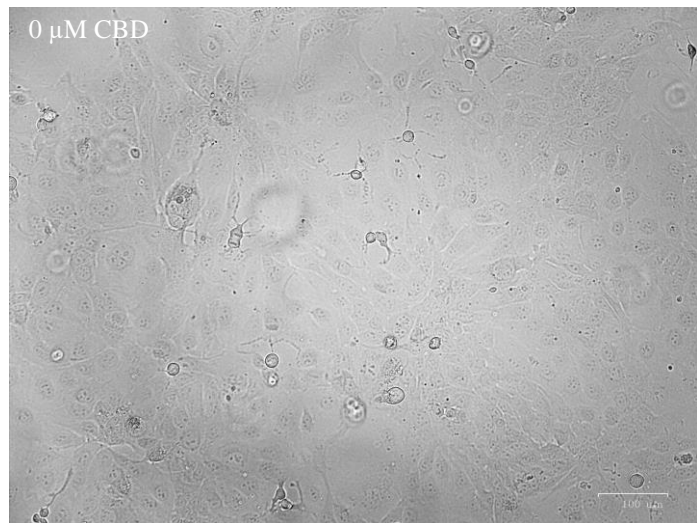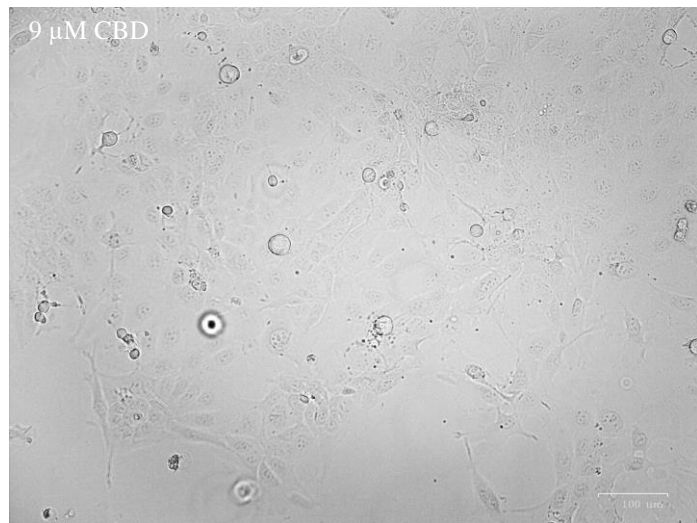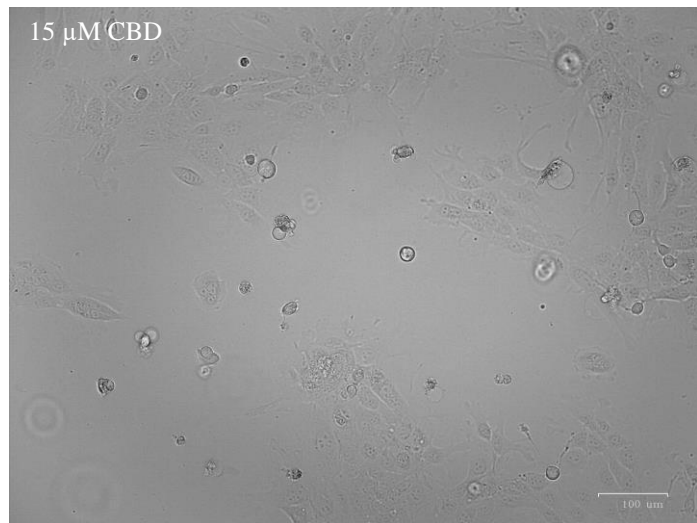

Supplement: Supplementary file 1 [file molecules-28-07887-s001.zip › Supplementary Figure S7 PNT CBD.pdf]

**10% FBS**

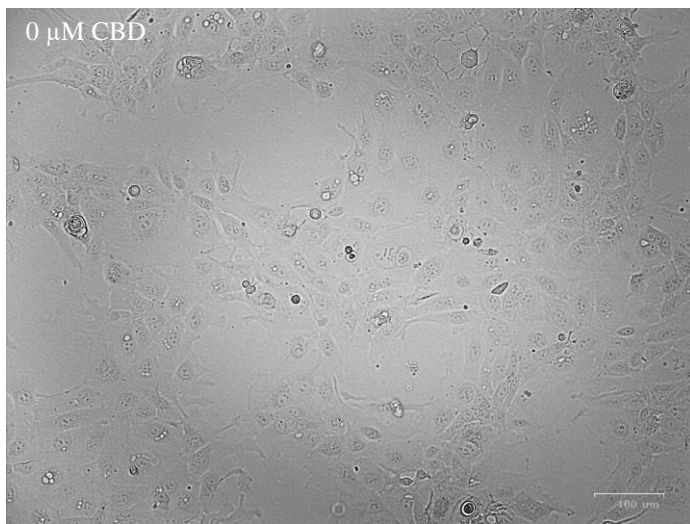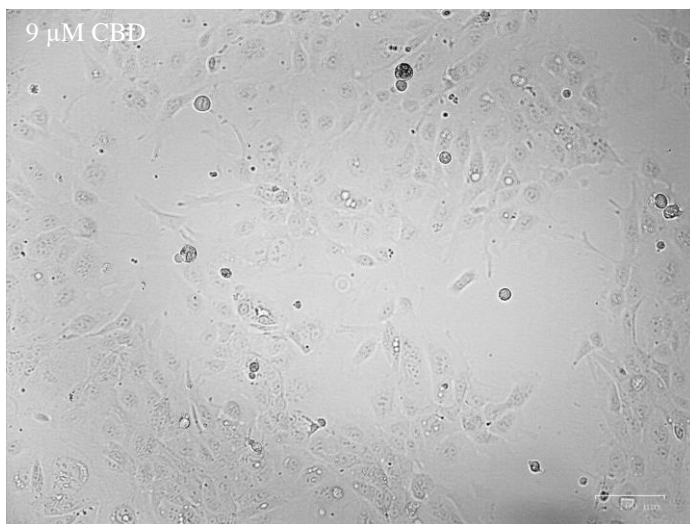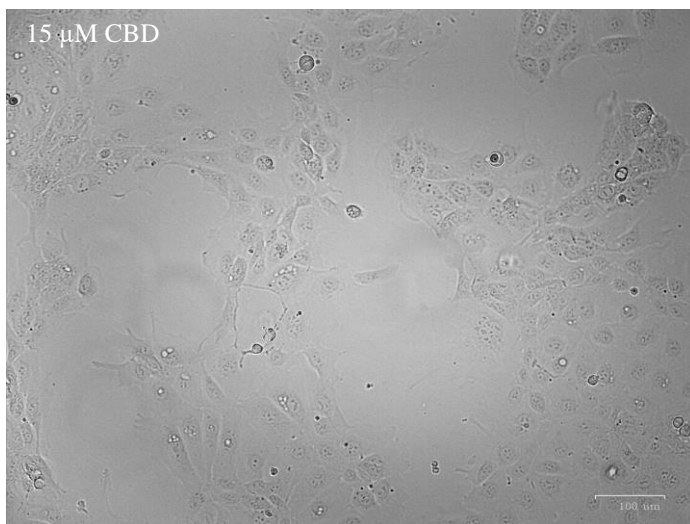

**0.5% FBS**

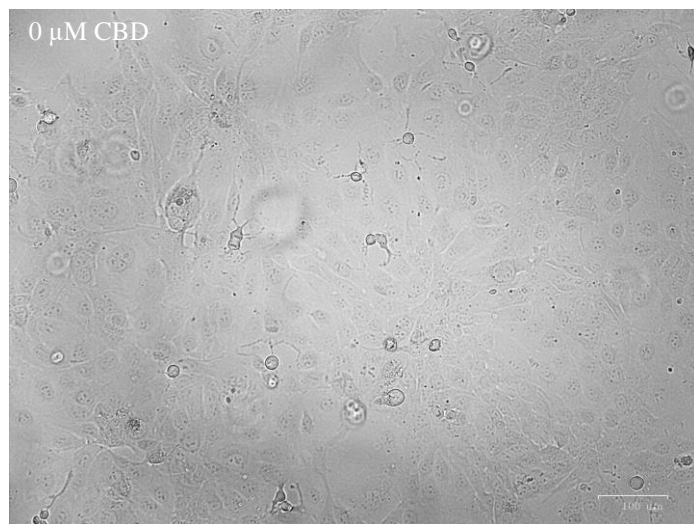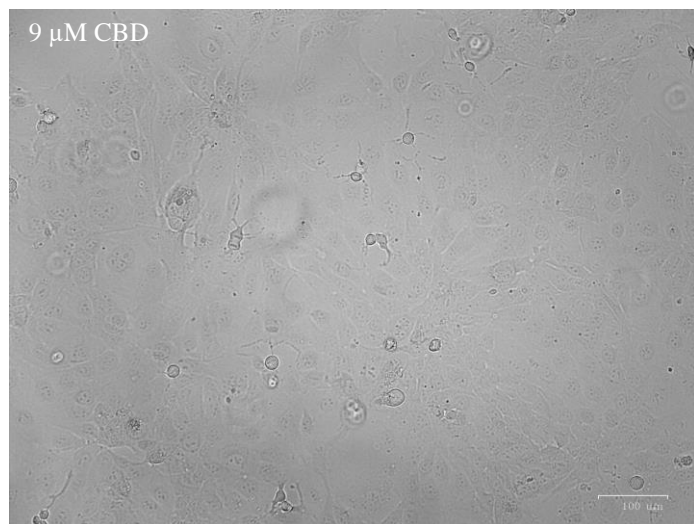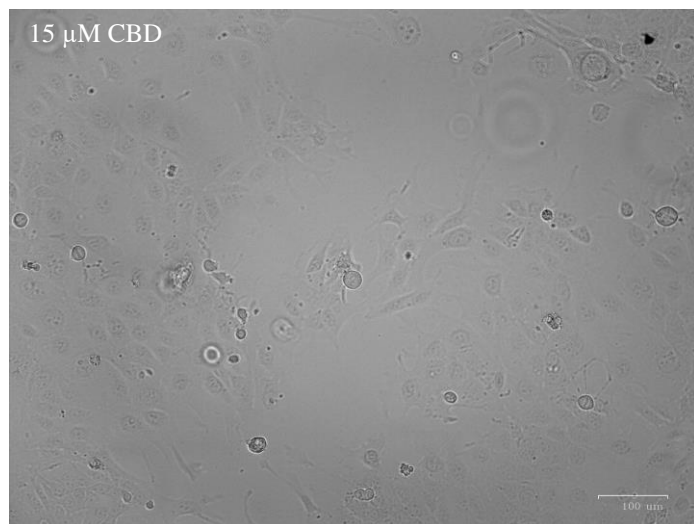

Supplement: Supplementary file 1 [file molecules-28-07887-s001.zip › Supplementary Figure S8 PNT EB.pdf]

## 10% FBS

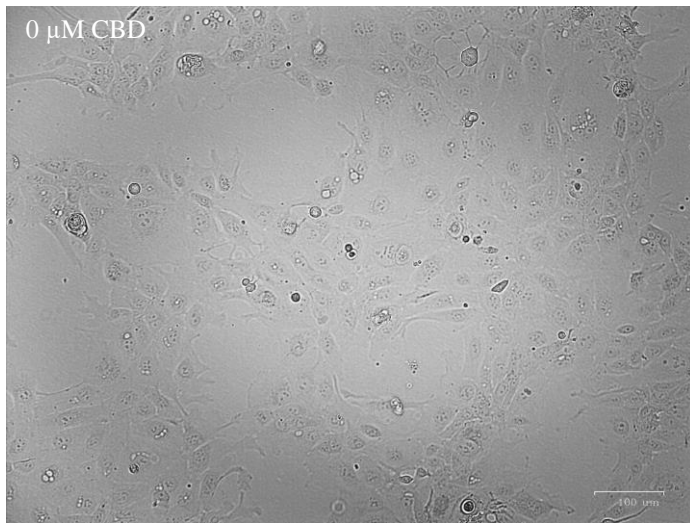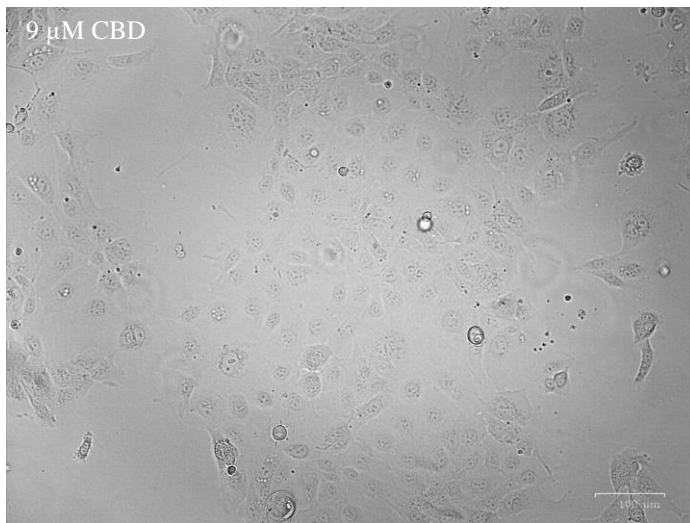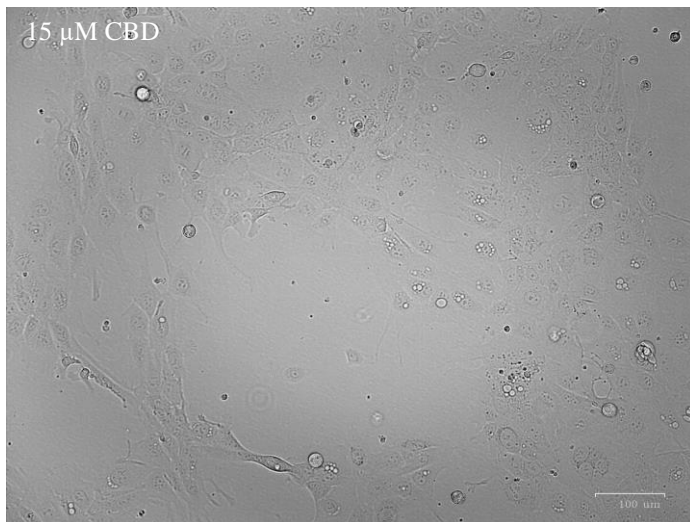

## 0.5% FBS

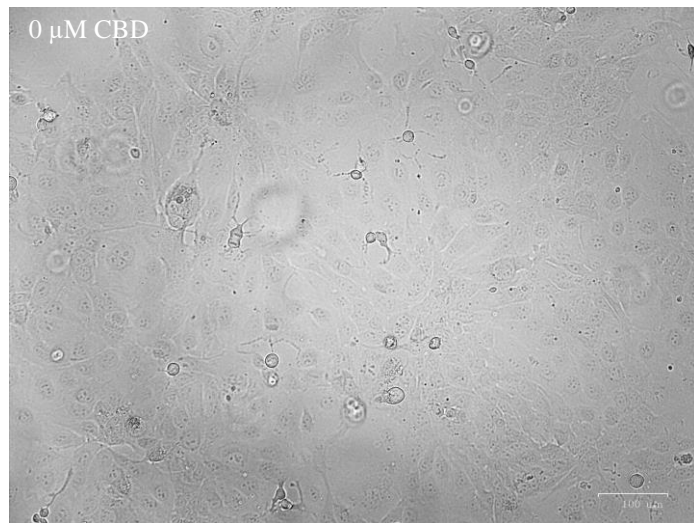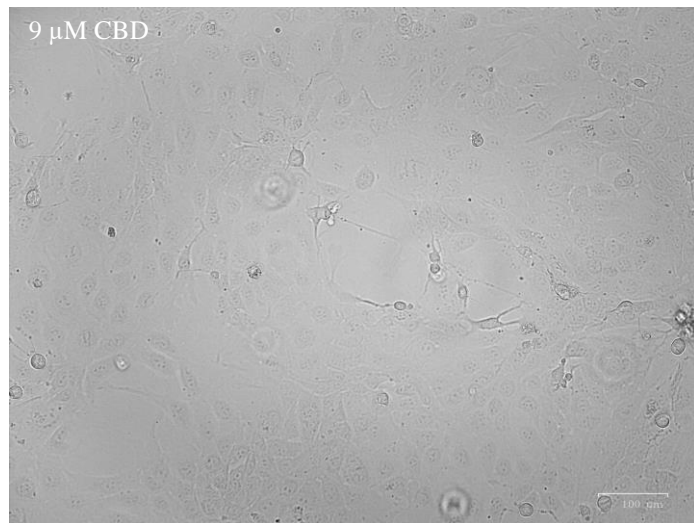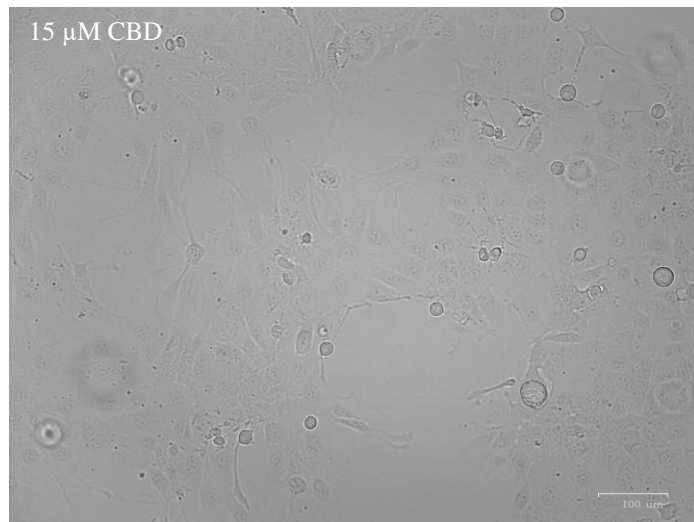

Supplement: Supplementary file 1 [file molecules-28-07887-s001.zip › Supplementary Figure S9 PNT ED.pdf]
